# Supplementary figures and images for: Characterization of the spatiotemporal representations of visual, semantic, and memorability features in the human brain
Source: PLoS Biol. 2026 Jan 20;24(1):e3003614. doi: 10.1371/journal.pbio.3003614 (PMC12851468; doi:10.1371/journal.pbio.3003614)

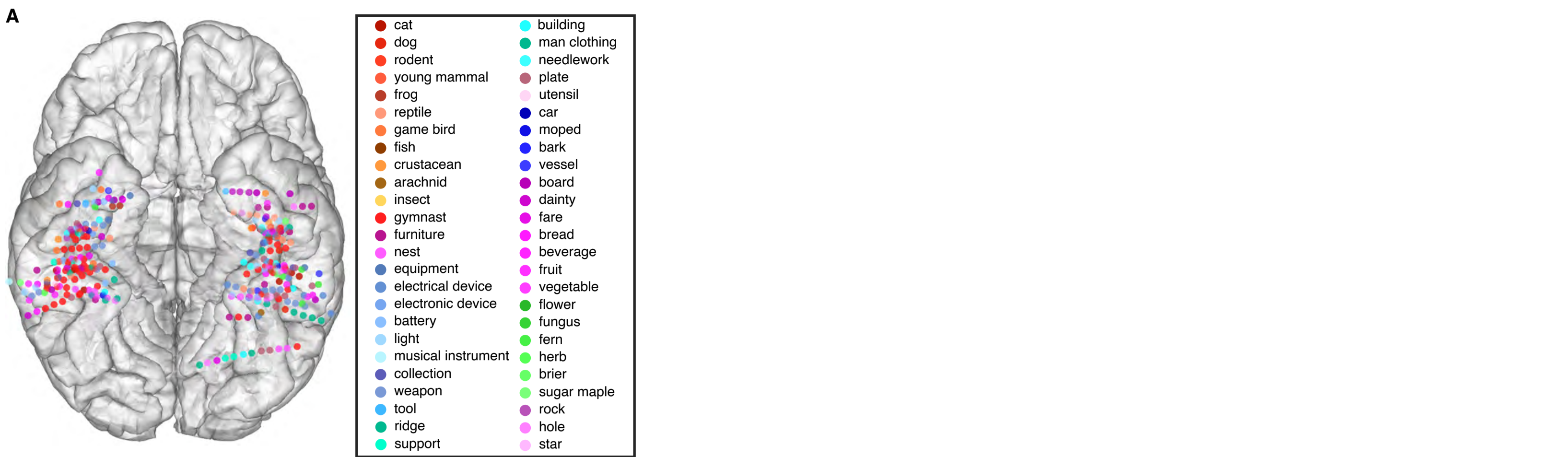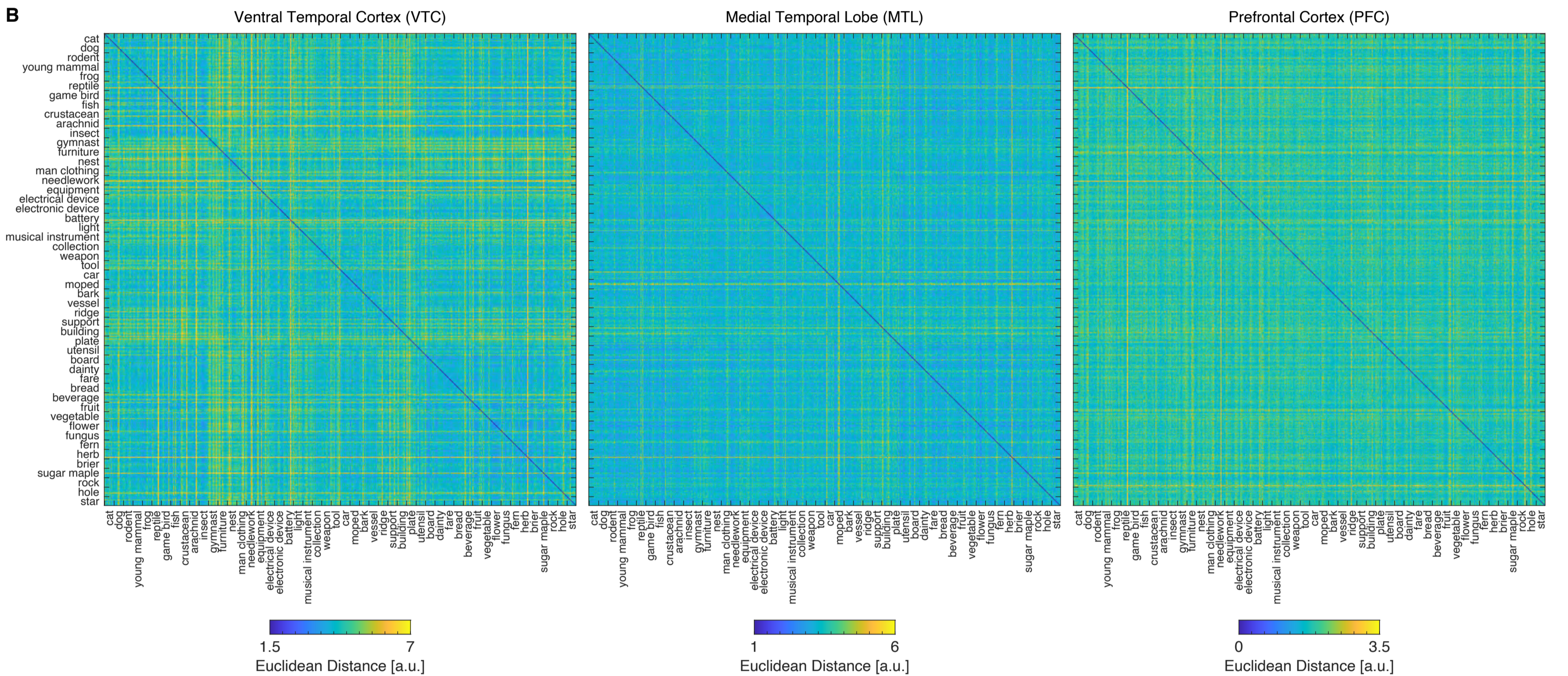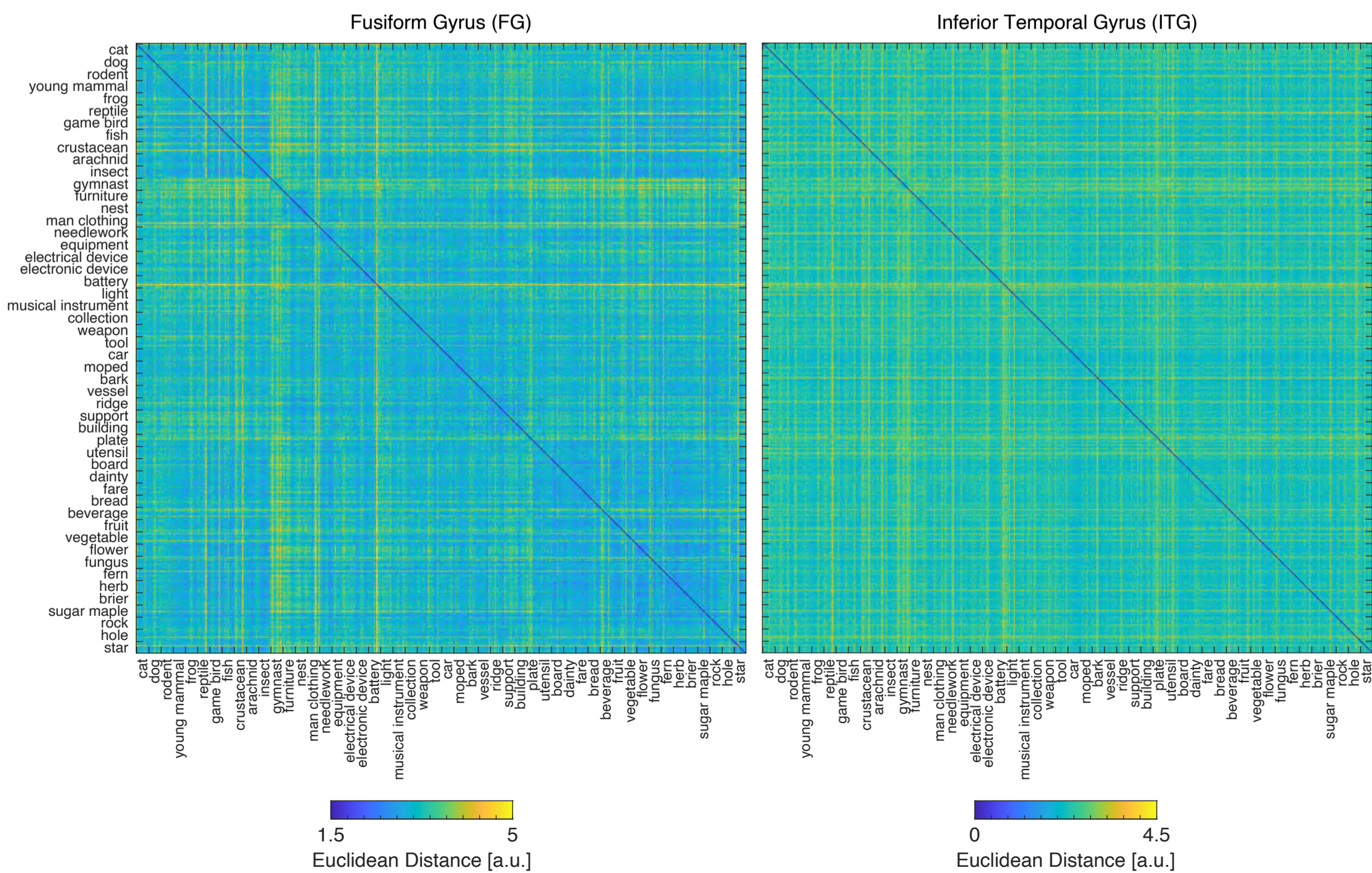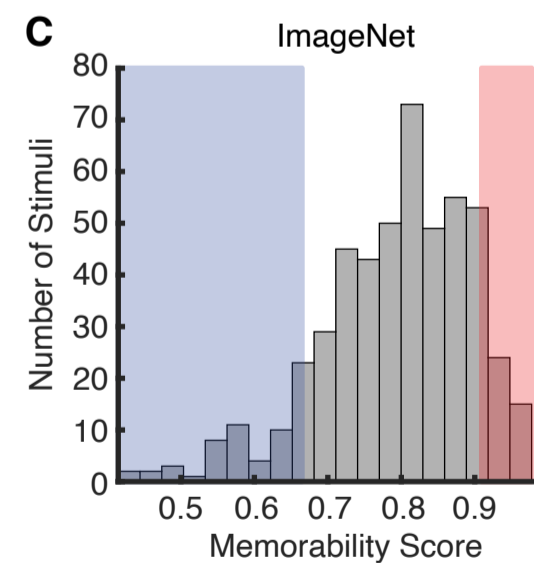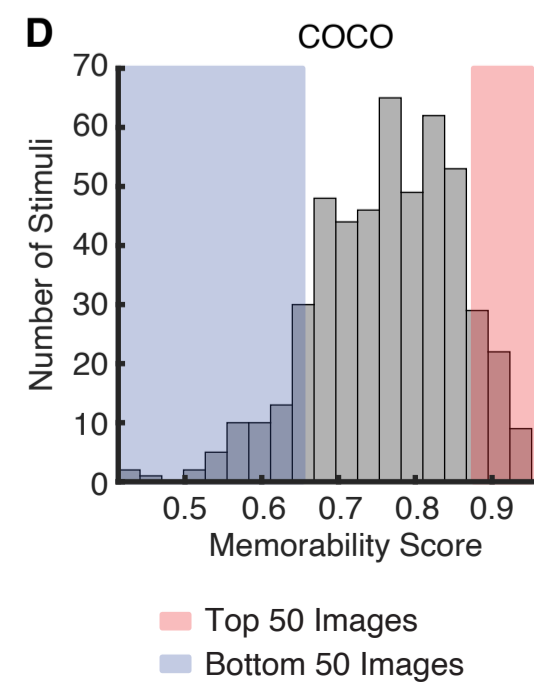

Supplement: S1 Fig — (A) Object category eliciting the maximal response for each visually responsive channel. Each color represents a different object category. (B) Neural RDMs of population responses for each ROI, sorted by object category. The RDMs were computed using all visually responsive channels within each ROI. (C) Distribution of memorability scores for the ImageNet stimuli. (D) Distribution of memorability scores for the COCO stimuli. The source data underlying this figure are provided in S7 Data. (PDF) [file pbio.3003614.s001.pdf]

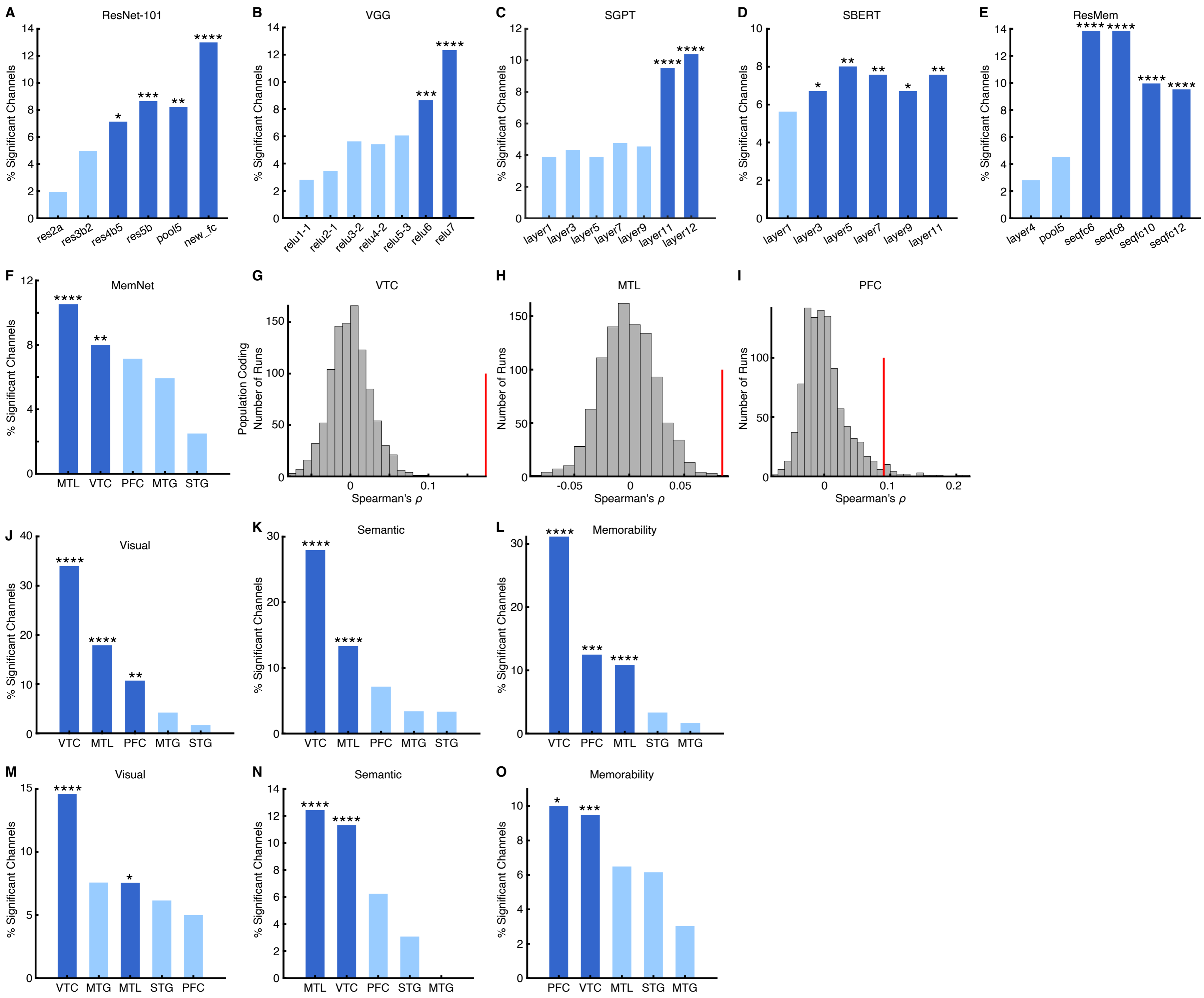

Supplement: S2 Fig — (A–E) Percentage of significant channels as a function of model layer, illustrated here using the ventral temporal cortex (VTC). (A) Encoding of visual features using the ResNet-101 model. (B) Encoding of visual features using the VGG model. (C) Encoding of semantic features using the SGPT model. (D) Encoding of semantic features using the SBERT model. (E) Encoding of memorability features using the ResMem model. (F) Percentage of significant channels in each region of interest (ROI) using the MemNet model. (G–I) Population-level neural encoding of (G) visual, (H) semantic, and (I) memorability features in the most prominent ROI for each attribute. RDMs were constructed using cosine distance. (J–L) Percentage of significant channels encoding (J) visual, (K) semantic, and (L) memorability features using linear models. (M–O) Percentage of significant channels encoding (M) visual, (N) semantic, and (O) memorability features using data from the first session of each participant. Legend conventions as in Fig 2. The source data underlying this figure are provided in S8 Data. (PDF) [file pbio.3003614.s002.pdf]
