# Supplementary material for: Characterization of the spatiotemporal representations of visual, semantic, and memorability features in the human brain
Source: PLoS Biol. 2026 Jan 20;24(1):e3003614. doi: 10.1371/journal.pbio.3003614 (PMC12851468; doi:10.1371/journal.pbio.3003614)
Supplement: S1 Table — VTC, ventral temporal cortex; MTL, medial temporal lobe; PFC, prefrontal cortex; FG, fusiform gyrus; ITG, inferior temporal gyrus; Lingual, lingual gyrus; LOC, lateral occipital cortex; Amy, amygdala; AH, anterior hippocampus; PH, posterior hippocampus; ERC, entorhinal cortex; PHC, parahippocampal cortex; SFG, superior frontal gyrus; POp, pars opercularis cortex; MFG, middle frontal gyrus. (PDF) [file pbio.3003614.s003.pdf]

ID	Sex	Age	Race	Epilepsy diagnosis	**Number of Channels**											
					**VTC**				**MTL**					**PFC**		
					**FG**	**ITG**	**Lingual**	**LOC**	**PH**	**AH**	**Amy**	**PHC**	**ERC**	**SFG**	**POp**	**MFG**
BJH024	F	38	Caucasian	Left temporal	13	17	0	0	5	3	7	2	3	7	4	4
BJH025	F	45	Caucasian	Right mesial temporal	12	18	0	0	5	2	14	2	5	6	2	2
BJH026	M	40	Caucasian	Left mesial temporal	14	13	0	0	1	0	8	1	2	0	2	6
					14	13	0	0	1	0	8	1	4	0	2	6
BJH027	M	37	Caucasian	Right temporal	16	37	0	0	5	10	9	2	4	0	2	3
					16	37	0	0	4	10	9	2	4	0	2	3
BJH028	F	36	Caucasian	Left frontal SMA, cingulate regions	0	0	0	0	0	0	3	0	0	19	4	16
BJH029	F	34	Caucasian	Left amygdala and hippocampus	15	18	6	2	5	10	10	2	4	0	0	0
					15	19	6	2	5	10	10	2	4	0	0	0
BJH030	F	22	Caucasian	Right amygdala and hippocampus	17	35	0	0	11	8	8	2	7	0	0	0
					17	35	0	0	15	4	7	2	7	0	0	0
BJH032	M	27	Caucasian/ American Indian	Bilateral amygdala and hippocampus	20	17	0	0	9	3	9	7	0	0	7	5
					20	17	0	0	9	3	8	6	0	0	7	5
BJH033	F	24	Caucasian/ American Indian	Right hippocampus	8	19	0	0	4	3	9	3	3	0	0	0
					11	21	0	0	5	4	9	3	3	0	0	0
BJH037	M	46	Caucasian	Bilateral amygdala and hippocampus	13	26	0	0	2	5	12	4	2	4	3	9
BJH050	F	28	Caucasian	Right temporal	15	15	0	0	4	8	7	2	1	2	0	8
BJH051	F	41	Caucasian	Left amygdala and hippocampus	9	11	0	0	2	5	9	6	3	1	0	6
BJH052	M	24	Caucasian	Bilateral hippocampus	16	22	0	0	4	8	9	10	2	2	8	0
					16	22	0	0	4	8	9	10	2	2	8	0
BJH053	F	20	Caucasian	Right middle frontal	1	2	0	0	0	1	3	0	0	16	5	26
BJH054	F	36	Caucasian	Left temporal	2	10	3	0	1	0	3	0	0	4	4	4
BJH056	F	36	Caucasian	Left amygdala and superior temporal gyrus	17	33	0	0	1	6	11	8	10	2	1	12
					17	33	0	0	1	6	11	8	10	2	1	11
BJH065	M	39	American Indian/ Alaskan Native	Right temporal	7	8	0	0	3	1	6	0	0	1	5	2
					8	8	0	0	3	1	6	0	0	1	5	2
BJH073	F	34	Caucasian	Right inferior temporal	4	0	0	0	0	5	0	0	0	0	13	14
BJH074	M	30	Caucasian	Right amygdala	6	19	0	0	3	3	11	1	6	12	1	16
					6	19	0	0	3	3	11	1	6	12	1	16
BJH075	M	25	Black/ African American	Left hippocampus	8	8	0	0	0	0	7	1	0	0	0	0
	**Sum**				353	552	15	4	115	130	243	88	92	93	87	176
