# Supplementary material for: Characterization of the spatiotemporal representations of visual, semantic, and memorability features in the human brain
Source: PLoS Biol. 2026 Jan 20;24(1):e3003614. doi: 10.1371/journal.pbio.3003614 (PMC12851468; doi:10.1371/journal.pbio.3003614)
Supplement: Attachment — Submitted filename: Response to Reviewers_PB_v4.pdf [file pbio.3003614.s012.pdf]

Reply to comments from Reviewer 1

In their submission, Wang et al. employ a multimodal approach to investigate the spatiotemporal brain dynamics associated with processing visual, semantic, and memorability features of naturalistic images in the human brain. Specifically, the authors recorded intracranial EEG (iEEG), including single unit data, during a simple 1-back visual recognition task. In addition, these data are complemented with open-source high-resolution 7T fMRI data, which employed a similar serial recognition task. Data analysis focused primarily on representational similarity analysis (RSA) across these different measurements, with reference to inter-stimulus correlation feature spaces derived from neural network estimates of visual, semantic and memorability features of the stimuli used. With this approach, the authors report that visual, semantic, and memorability features are dissociable in distinct brain regions —VTC, MTL, and PFC—with different temporal profiles. Memorability representations in PFC appear to arise from integrated visual and semantic signals in the VTC and influence early perceptual processing. However, the PFC data appears somewhat underpowered, and the subtle timing differences across regions are unexpected. Overall, the work brings together important findings regarding the complex neural interactions, beyond sensory detection, that must occur during successful recognition, but some clarification is required to fully appreciate the advance provided.

We thank the reviewer for the expert and constructive comments.

Major:

-PFC: Overall, the results related to PFC contain few electrodes (10 or 6) and was not significant in the linear decoding analysis for memorability. Looking at the anatomical plots, it also appears these are electrodes from a few common probes, suggesting the effect in PFC is likely driven by only a few subjects. How were multiple observations coming from subjects controlled e.g. mixed or nested effects analysis (RSA or linear decode analyses)? Also, anatomically, it's not clear why the interpretation shifts to superior lateral frontal (SLF), given the locations of the electrodes shown. The authors should clarify how nested data is being handled, and how many subjects show significant effects in PFC, with interpretation recalibrated to the actual strength/consistency of this finding.

We thank the reviewer for pointing this out. The reviewer is correct in noting that in our previous manuscript, only a few electrodes in the PFC (n = 10) showed significant memorability-related responses. However, we would like to clarify that our analysis was limited to stimulus-responsive channels, and the total number of such channels in the PFC was relatively small (n = 83). To address this concern, we have now included six additional patients, contributing 59 new PFC channels and appreciably increasing our statistical power. In the revised manuscript, we also adjusted the PFC region of interest (ROI) to focus on subregions within this very broad brain area that exhibit stronger visual responsiveness. As a result, we now observe significant linear decoding in the PFC for memorability (**Fig. S2L**).

Second, we found that 7 out of 20 patients contributed significant channels, indicating that the results were not solely driven by individual differences. Notably, we performed a bootstrapping test to account for participant-level effects. In each of 1000 iterations, we randomly subsampled PFC channels from 15 patients (75%) and calculated the percentage of memorability-selective channels. The observed percentage did not significantly differ from the bootstrapped distribution ($P = 0.18$), suggesting that our results were not solely driven by participant-specific effects. We further confirmed this finding using a general linear mixed-effects (GLME) model. Specifically, we compared a full model—including participant as a random effect—with a reduced model excluding the random effect. A likelihood ratio test revealed that the random effect of participant was not significant ($P > 0.05$), reinforcing the robustness of our findings across participants.

We have updated the results in the revised manuscript:

"Twenty neurosurgical patients (12 females; **Table S1**) undergoing intracranial monitoring with implanted depth electrodes participated in this study. We recorded iEEG activity while participants viewed 500 ImageNet images [1] (see **Fig. S1A** for examples), in which they were instructed to respond whenever an image was repeated in two consecutive trials (**Fig. 1A**; see **Methods** for details). Participants demonstrated high task engagement, with a mean accuracy of 87.53% ± 10.82% (mean ± SD) across sessions.

We recorded iEEG from a total of 5143 channels (**Fig. 1B**), covering the VTC (n = 924), superior temporal gyrus (STG; n = 551), middle temporal gyrus (MTG; n = 565), MTL (n = 668), and PFC (n = 356). These brain areas were designated as regions of interest (ROIs). For analysis, we extracted high-

gamma power (HGP; 70–170 Hz) from each iEEG channel, as HGP reflects the average neuronal firing of local neural populations [2, 3]. We identified a total of 1678 visually responsive channels (black dots in **Fig. 1B**) by comparing neural responses around peaks (0.1 to 0.6 seconds relative to stimulus onset; shaded area in **Fig. 1C**) to baseline activity (−0.5 to 0 seconds relative to stimulus onset), including 462 channels in the VTC, 120 channels in the STG, 118 channels in the MTG, 285 channels in the MTL, and 112 channels in the PFC. Subsequent analyses were restricted to these visually responsive channels."

And:

"We observed a significant percentage of channels encoding visual features in the VTC (n = 63, 13.64%, binomial test against 5% chance level: P = 3.74×10^{-13}; **Fig. 2A, B**), semantic features in the VTC (n = 51, 11.04%, binomial P = 6.24×10^{-8}) and MTL (n = 31, 10.88%, binomial P = 1.99×10^{-5}; **Fig. 2E, F**), and memorability features in the PFC (n = 12, 10.71%, binomial P = 0.004), MTL (n = 22, 7.72%, binomial P = 0.017), MTG (n = 10, 8.47%, binomial P = 0.035), and VTC (n = 32, 6.93%, binomial P = 0.035; **Fig. 2I, J**). Notably, our results held after accounting for individual differences using both a bootstrapping test and a generalized linear mixed-effects model (GLME; both Ps > 0.05; see **Methods** for details)."

And:

"The results were qualitatively consistent with the RDM model, as we observed robust encoding of visual (**Fig. S2J**; n = 157, 33.98%, binomial P = 7.14×10^{-14}), semantic (**Fig. S2K**; n = 129, 27.92%, binomial P = 7.13×10^{-14}), and memorability (**Fig. S2L**; n = 144, 31.17%, binomial P = 7.13×10^{-14}) features in the VTC. The percentage of selective channels identified by the linear model was even significantly higher than those identified by the RDM model (χ^2-test; visual: P = 3.85×10^{-13}; semantic: P = 9.23×10^{-11}; memorability: P < 1×10^{-20}). Furthermore, we observed a significant number of channels encoding visual (n = 51, 17.89%, binomial P = 1.41×10^{-13}), semantic (n = 38, 13.33%, P = 1.55×10^{-8}), and memorability (n = 31, 10.88%, binomial P = 1.99×10^{-5}) features in the MTL; and we also observed a significant number of channels encoding visual (n = 12, 10.71%, binomial P = 0.004) and memorability (n = 14, 12.5%, binomial P = 4.79×10^{-4}) features in the PFC."

And we have included the following methods in the revised manuscript:

"*Control analysis for individual differences*

To rule out the possibility that our results were driven by specific participants, we performed a participant-level bootstrapping test and applied a generalized linear mixed-effects (GLME) model, treating participant as a random effect, for the selection of channels representing visual, semantic, and memorability features. In the bootstrapping test (1000 iterations), we randomly subsampled PFC channels from 15 patients (75% of the total sample) and calculated the percentage of selective channels in each iteration. If the observed percentage of selective channels fell within the bootstrapped distribution, this would suggest that the effect was not solely driven by individual participants. In the GLME analysis, we fit a full linear model with participant included as a random effect (percentage ~ feature type + (1 | participant)) and compared it to a reduced model without the random effect (percentage ~ feature type). We then conducted a likelihood ratio test to determine whether including the random effect significantly improved model fit."

Third, we apologize for the confusion. In the replication study using the COCO stimuli, we observed significant channels primarily in the superior lateral frontal (SLF) cortex; therefore, we used this label to refer to the PFC. We now present the results using channels across the PFC, applying the same ROI as in the main study. We have also updated our results in the manuscript:

"Consistent with our earlier results, we confirmed the encoding of visual features in the VTC (n = 33, 12.27%, binomial P = 8.15×10^{-7}) and MTG (n = 5, 9.62%, binomial P = 0.04; **Fig. 5A, B**); semantic features in the MTL (n = 17, 10.24%, binomial P = 0.0018) and VTC (n = 25, 9.29%, binomial P = 0.0011; **Fig. 5E, F**); and memorability features in the VTC (n = 29, 10.78%, binomial P = 4.02×10^{-5}), PFC (n = 7, 9.33%, binomial P = 0.033), and MTL (n = 15, 9.04%, binomial P = 0.0094; **Fig. 5I, J**). Furthermore, the VTC, MTL, and PFC represented the corresponding visual (**Fig. 5C**; Spearman's ρ = 0.17, permutation P < 0.001), semantic (**Fig. 5G**; Spearman's ρ = 0.15, permutation P < 0.001), and memorability (**Fig. 5K**; Spearman's ρ = 0.08, permutation P < 0.001) features, respectively, at the population level. Similarly, visual encoding in the VTC peaked first (**Fig. 5D**; 290 ms), followed by semantic encoding in the MTL (**Fig. 5H**; 450 ms), and memorability encoding in the PFC (**Fig. 5L**; 450 ms)."

Fourth, we would like to clarify that two recording sessions were conducted for a subset of patients. As a result, channels from different sessions of the same patient may overlap anatomically. To control for potential confounds arising from varying numbers of sessions across patients, we replicated our results

using only the first session from each participant (**Fig. S2M-O**). We have included the following results in the revised manuscript:

“Furthermore, to account for the fact that a subset of patients underwent two recording sessions (**Table S1**), we repeated all analyses using only the first session from each participant and obtained consistent results (**Fig. S2M-O**).”

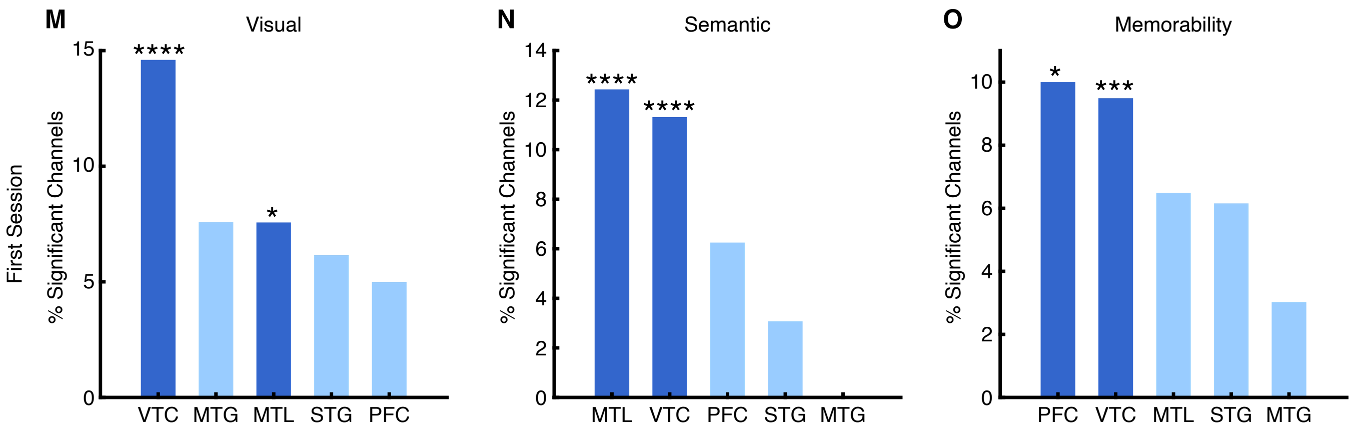

Fig. S2. (M–O) Percentage of significant channels encoding **(M)** visual, **(N)** semantic, and **(O)** memorability features using data from the first session of each participant.

Finally, we have revised our description of the results to tone down claims about the specific engagement of the PFC in memorability encoding, as follows:

“Together, by examining the neural correlates of these three attributes collectively and comprehensively, we revealed their differential distributions in the human brain: visual information is represented primarily in the VTC, semantic information is represented mainly in the VTC and MTL, and memorability processing engages a broader distribution of higher-level brain areas beyond the VTC.”

We have also revised the **Abstract** as follows:

“Here, we recorded intracranial electroencephalography from 5143 channels while participants viewed natural object images. We systematically characterized the spatiotemporal patterns of neural encoding for visual, semantic, and memorability attributes and showed that memorability was encoded in a distributed manner, which can be dissociated from visual and semantic coding.”

-Response timing: Latency of response times are strikingly similar across regions, despite there being a wealth of data (including human iEEG) that consistently shows expected differences. High-gamma response examples in Figure 1c appear to be highly similar, as do the timing peaks in Figure 2d,h,l, and Figure 3c. However, a lot of data shows VTC response times (typically 100-200ms) are consistently earlier than MTL/hipp (300+ ms), so there is some concern about the anatomical inclusion criteria that are making responses all appear around the same time. I appreciate that some of these analyses are related not just to the raw response but to feature correlations, but prior work has shown such approaches also conform to known timing differences through the ventral stream. The authors should provide mean spectrograms or high-gamma plots, for these anatomical regions of interest to show the data going into RSA analyses is consistent with prior observations. This is also important as many analyses use electrode counts, which is removing all magnitudes and compresses variance and is not a sensitive means for capturing consistent response features. Otherwise, the author's opening point about the stages of recognition is in contrast to reporting mostly simultaneous events across cortex during recognition.

We thank the reviewer for pointing this out and we agree with the reviewer. The response latency noted by the reviewer has been consistently observed in our previous studies [4-9] (note that the values mentioned refer to *onset* latencies rather than *peak* latencies reported in our manuscript; see [10] for a comprehensive analysis), and we are fully aware of the latency differences between the VTC and MTL.

First, we would like to clarify that the measure of onset latency is relative. Depending on the statistical threshold and/or the size of the moving window, the measured onset latency may vary; therefore, the exact numbers may differ across studies. For this reason, we focused on comparing peak latency, which is more stable across different criteria. We have now labeled the peak latencies on the time course of high-gamma responses in **Fig. 1C**, as well as on the RSA correlations in **Fig. 2D, H, L** and **Fig. 5D, H, L**. As long as the same measurement approach is applied, latency can be meaningfully compared across brain regions.

Indeed, we found that "the encoding of visual features peaked at 350 ms in the VTC, followed by semantic encoding in the MTL at 410 ms, and memorability encoding in the PFC at 410 ms", suggesting a hierarchical processing sequence. This pattern was more prominent in the COCO dataset, where visual feature encoding peaked at 250 ms in the VTC, followed by semantic encoding in the MTL at 450 ms

and memorability encoding in the PFC at 450 ms. In addition, consistent with this interpretation, onset latency in the VTC was earlier than in the MTL when calculated using the same criteria, as shown in the manuscript.

We acknowledge that the correlation peak in the VTC may appear later than expected, which reduced the latency difference between the VTC and MTL. This was likely due to parallel processing of visual, semantic, and memorability feature information, as multiple routes of information flow may exist [11]. Similarly, the comparable latencies for the representations of the three types of information shown in **Fig. 3C** may also reflect parallel processing. Nevertheless, these results are generally consistent with our previous observations [4-9].

We have also provided HGP plots for the data used to compute RSA in **Fig. 2D, H, L** (**Revision Fig. 1**). The response profiles are consistent with the extensive human iEEG literature (e.g., [11, 12]).

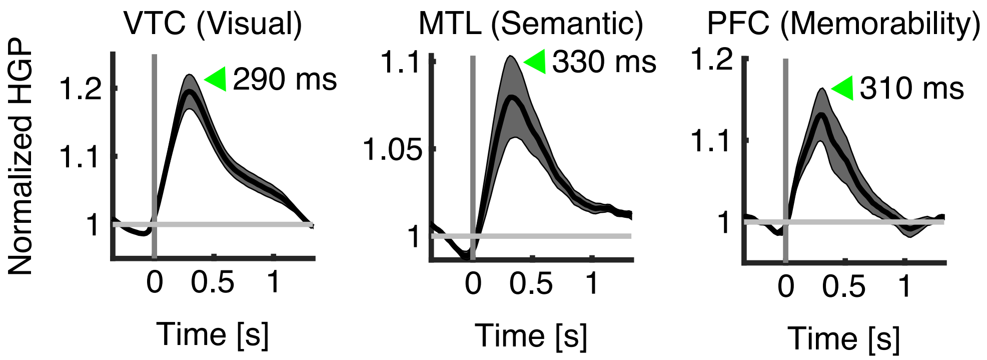

Revision Fig. 1. High-gamma power (HGP) in the VTC, MTL, and PFC for channels involved in RSA computation. Legend conventions as in **Fig. 1C**.

-RSA analyses: The crucial analysis in this manuscript is the comparison of high gamma responses to the output layers from different neural network models. That means that any interpretation of the results presented here is heavily reliant on the interpretation of the representations encoded in these layers. As such more space could be devoted to explaining why these particular layers of these specific models were chosen and what manner of validity the layers have to their constructs (especially in regards to the memorability features). Could the authors provide more detail, perhaps expanding on the visualization in Figure 1d, about the relationship between model layers and the constructs they represent? Similarly,

could the authors provide information about how robust these results are to the specific models/methods used (e.g. the choice of Euclidean distance vs. cosine distance, or using the last layer of ResMem instead of MemNet)?

We thank the reviewer for raising this important question. We agree that the interpretation of our findings depends in part on understanding the representational properties of the neural network layers we analyzed. In the revised manuscript, we demonstrated the robustness of our results across different layers and models (**Fig. S2**). We have also expanded our explanation to clarify both our rationale for selecting these specific output layers and the validity of these layers in relation to the constructs of interest, particularly memorability.

First, we chose the output layers of each model because these layers contain the most task-relevant representations, reflecting the final transformation of input information that is most predictive of behavioral or semantic outputs. For example, in models trained on object classification, the output layer reflects high-level categorical or semantic features, while in models trained on memorability prediction (e.g., ResMem), the output is explicitly optimized to represent features predictive of human memory performance. Therefore, these layers are the most appropriate for evaluating how well model-derived features align with neural activity associated with corresponding cognitive functions, especially memory encoding. Notably, we demonstrated that our results were robust across later layers for each feature type (**Fig. S2**).

Second, we selected models that have been previously validated in the literature for their ability to predict memorability or related constructs. For instance, ResMem was trained and evaluated directly on large-scale human memorability ratings, and its output layer has been shown to correlate with memory performance across large image sets and participant populations [12]. We chose to use ResMem rather than MemNet because the former was trained on a larger dataset combining both the LaMem [13] and MemCat [14] datasets, whereas the latter was trained only on LaMem. SGPT, although trained for image-text alignment, has been shown to capture semantically rich representations that generalize across a wide range of cognitive and perceptual tasks, including aspects of memory [15]. These prior validations support the interpretability and construct validity of the representations encoded in the final layers of the models we used. To further validate our findings, we replicated the results using an additional established model for each feature type (**Fig. S2**). Specifically, we found similar results for

the representation of visual features using VGG [16], semantic features using Sentence-BERT [17], and memorability features using MemNet [18]. These results have been included in the revised manuscript:

“In addition, our results remained robust across different layers of the same model (**Fig. S2A, C, E**), as well as across different models (**Fig. S2B, D, F**). We also obtained consistent results using cosine distance rather than Euclidean distance (**Fig. S2G-I**).”

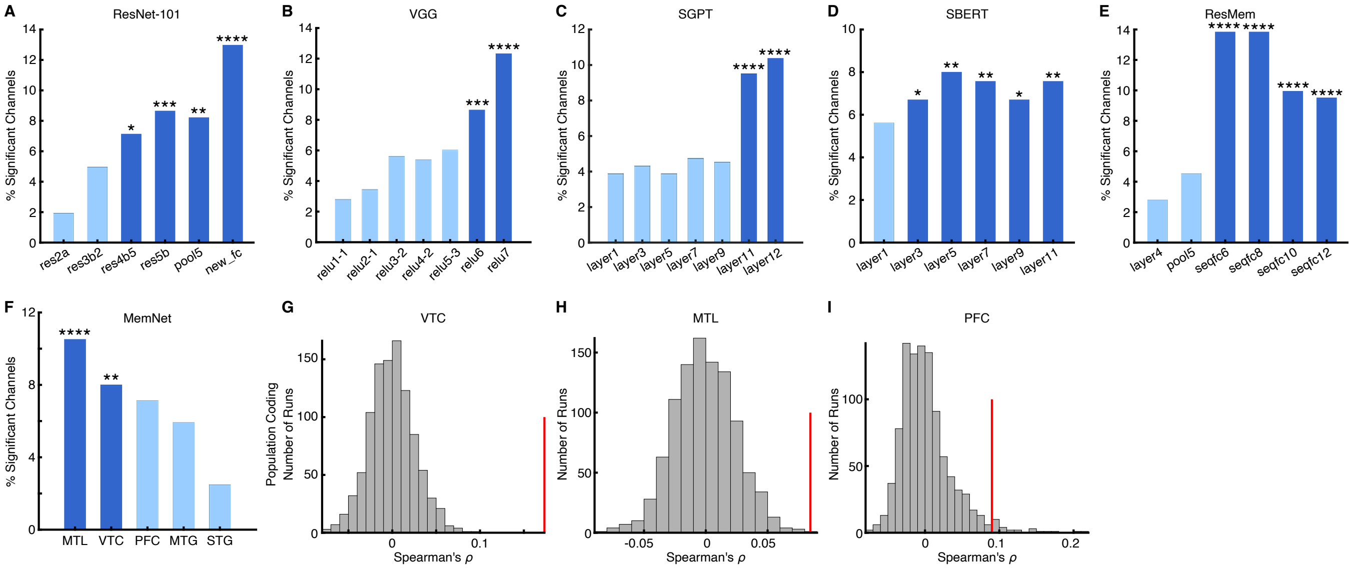

Fig. S2. Control analyses for the encoding of visual, semantic, and memorability features. (A–E) Percentage of significant channels as a function of model layer, illustrated here using the ventral temporal cortex (VTC). **(A)** Encoding of visual features using the ResNet-101 model. **(B)** Encoding of visual features using the VGG model. **(C)** Encoding of semantic features using the SGPT model. **(D)** Encoding of semantic features using the SBERT model. **(E)** Encoding of memorability features using the ResMem model. **(F)** Percentage of significant channels in each region of interest (ROI) using the MemNet model. **(G–I)** Population-level neural encoding of **(G)** visual, **(H)** semantic, and **(I)** memorability features in the most prominent ROI for each attribute. RDMs were constructed using cosine distance.

We have expanded the justification for our selection of models and layers used in constructing the different feature representations:

“We constructed representational dissimilarity matrices (RDMs) for visual, semantic, and memorability features using well-established and advanced models. For visual features, we used the pre-trained

ResNet [19] model for ImageNet stimuli and the AlexNet [20] model for COCO stimuli. Both models have demonstrated strong performance in object categorization for these datasets and have been shown to effectively model neural responses [21-23]. Following established procedures [8, 24], we fine-tuned the top layer of each model to verify that the pre-trained models could effectively discriminate object categories, thereby validating their suitability as feature extractors. For semantic features, we first used the ALBEF model [25] to generate text descriptions for each image. These descriptions were then processed using the SGPT model [15] to extract sentence embeddings. SGPT was pre-trained for image-text alignment and has been shown to capture semantically rich representations that generalize across a wide range of cognitive and perceptual tasks, including aspects of memory [15]. For memorability features, we employed the ResMem model [12], which was trained and evaluated directly on human memorability ratings across large image sets and participant populations [12, 26], to extract features predictive of image memorability.

In selecting feature representations from each model, we used the output layer as the feature extraction layer, with the exception of the AlexNet model, where we employed the FC6 layer in line with prior literature [21]. This choice was guided by the rationale that output layers contain the most task-relevant and abstracted representations, reflecting the culmination of computations optimized for specific behavioral or semantic objectives. For instance, in object classification models such as ResNet and AlexNet, the output layers encode high-level categorical or semantic structure, while in the ResMem model, the output is explicitly trained to predict human memory performance. Consequently, these layers are well-suited for assessing the alignment between model-derived features and neural activity associated with related cognitive functions, particularly memory encoding. Importantly, we confirmed that our main findings were robust across alternative higher-level layers for each feature type (**Fig. S2A-E**), supporting the generalizability of our results beyond specific layer choices."

Minor:

-Figure 3A is a little counterintuitive in how different data is being conveyed, with numbers not matching chart ratios.

We thank the reviewer for pointing this out and apologize for the confusion. We plotted only the significant channels, which is why the numbers did not match the chart ratios. We have now clarified this in the revised **Fig. 3A**.

- The direct comparison of fMRI and electrophysiology results shown in the first row of Figure. 4 are compelling. However, I'm less convinced about the utility of the analyses shown in the bottom row. For example, the analysis comparing coding strength between coding electrodes that do or do not overlap with the fMRI masks (K,N,Q, T), seems a bit circular in nature (electrodes that encode features have greater coding strength than those that do not). Also, the % iEEG channel overlap findings seem ambiguous, supporting electrophysiology results in the FG only, making it difficult to interpret the reliability of those results.

We thank the reviewer for pointing this out, and we agree with the comment. We would like to clarify that these results are *confirmatory* rather than *predictive*, aiming to validate findings within the significant fMRI voxels. We have noted in the revised manuscript that these results are as expected.

Furthermore, we would like to clarify that our analyses focused specifically on the FG and MTL due to the extremely high voxel count in the 7T dataset. Applying all models—particularly those involving computationally intensive permutation tests (1000 iterations)—across the entire brain would have been too time-consuming and computationally demanding. As a result, we limited our analysis to these regions of primary interest to ensure feasibility.

- For figure 6, it would be informative to show the waveforms for the example neurons. Also, did the authors have any criteria for including or excluding detected waveforms that could be described in the methods?

We thank the reviewer for the suggestion. We have now included the waveform for each example neuron in the revised figure (**Fig. 6J**).

J

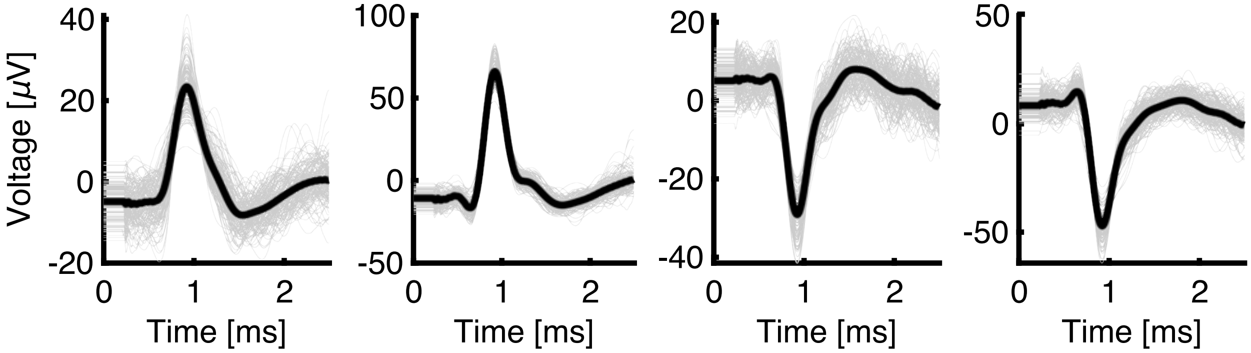

During spike sorting, we manually removed clusters with poor waveforms that did not resemble neuronal activity. We have clarified this in the revised manuscript:

<u>"During spike sorting, we manually removed clusters with poor waveforms that did not resemble neuronal activity."</u>

- For the fMRI analyses, why were trials with beta values <0 excluded?

We thank the reviewer for pointing this out. We apologize for the incorrect description—in fact, we did not exclude trials based on the polarity of beta values. We have removed this sentence from the manuscript.

- I'm curious about how to interpret the findings of VTC semantic representation. In particular, it would be useful to understand how the RDMs used here may relate to classical views of categorical selectivity in the VTC. Do the fusiform RDMs (or simply univariate responses), for example, demonstrate responsivity that may reflect some element of face selectivity?

We thank the reviewer for the question. The RDMs used in our analysis illustrate the structure of relationships between neural responses to different visual categories. To provide a more intuitive visualization, we rearranged the object categories in the RDMs based on superordinate labels (e.g., animals, insects, man-made objects, equipment, vehicles, food, plants, and natural objects; **Fig. S1C**). This revealed weak block-like patterns in the VTC, where images from the same category evoked more

similar neural responses—indicating categorical structure in these neural populations. For example, animal images (top left) were more similar to each other (i.e., lower dissimilarity values) than to other categories; and this was especially the case in the FG. However, we would like to clarify that the RDMs do not reflect preferential responses to specific categories such as faces, as they capture the relative distances between categories rather than direct comparisons of response amplitudes. Additionally, our image set did not include a category explicitly designated for face images (faces were primarily present in the "gymnast" category).

Despite this, we demonstrated classical category selectivity using a univariate analysis, in which we identified the most responsive category for each visually responsive channel (**Fig. S1B**; see [27] for more detailed results). Consistent with previous studies [28, 29], channels in the right FG responded most strongly to images from the gymnast and animal categories (highlighted in red), suggesting a semantic-based topography in the VTC.

Together, these additional results demonstrate that the VTC, especially the FG, exhibits structure consistent with known face- and category-selective responses. We have incorporated these results in the revised manuscript:

"Furthermore, we qualitatively observed that fusiform gyrus (FG) channels responded more strongly to human images (in the "gymnast" category) and animal images (**Fig. S1B**), consistent with classical views of visual category selectivity in the VTC [28-30] (note that population responses in the VTC also reflected categorical structure; **Fig. S1C**)."

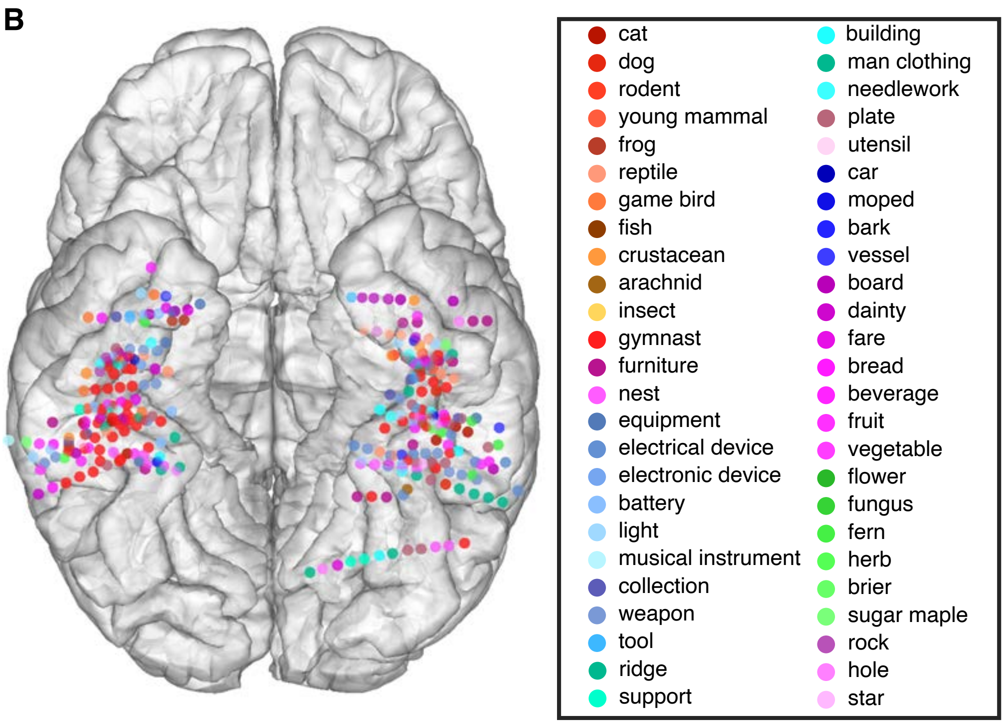

Fig. S1. (B) Object category eliciting the maximal response for each visually responsive channel. Each color represents a different object category.

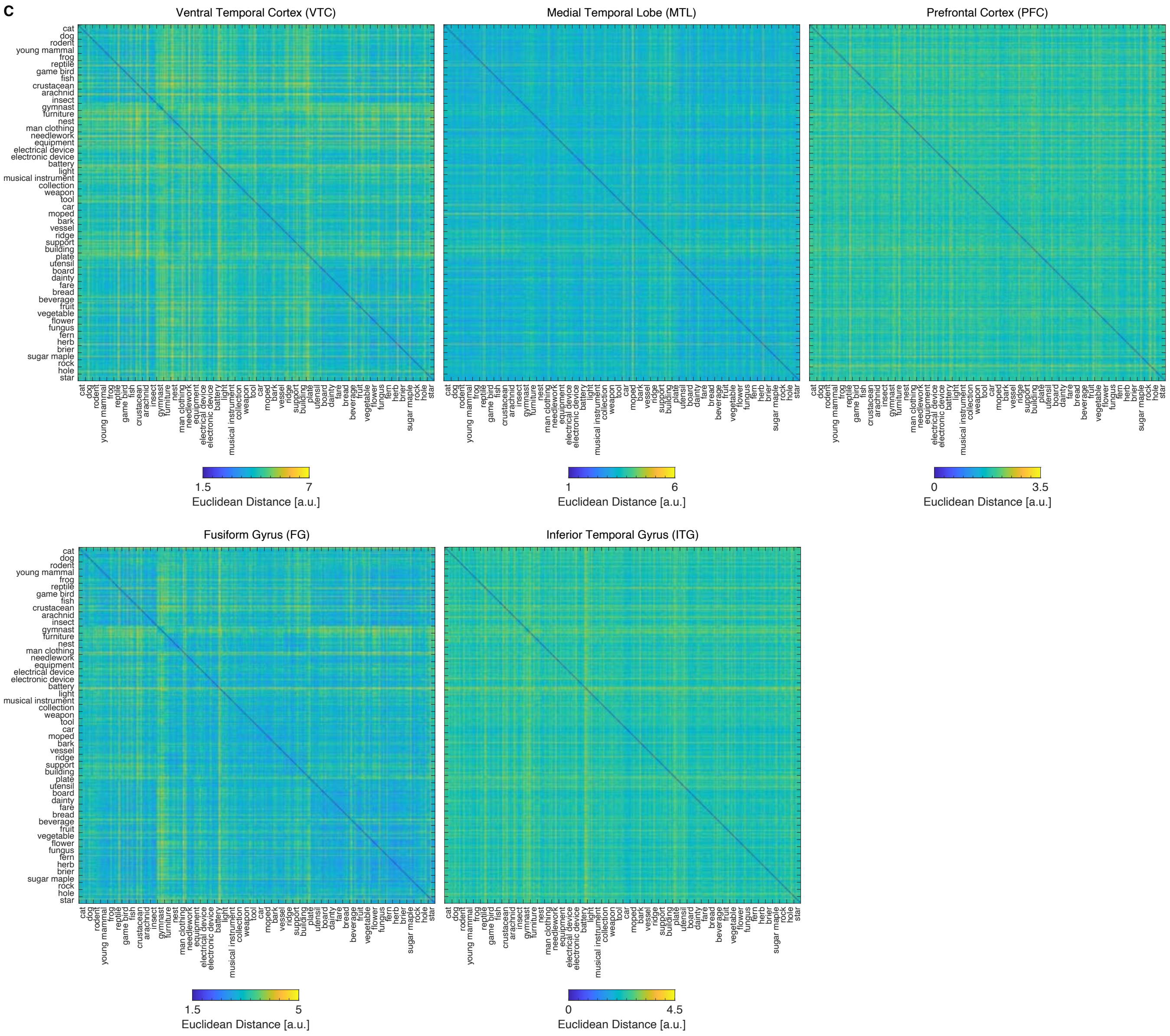

Fig. S1. (C) Neural RDMs of population responses for each ROI, sorted by object category. The RDMs were computed using all visually responsive channels within each ROI.

- On page 5, could the authors provide some quantitative backing to the statement 'qualitatively channels encoding semantic and memorability features were located more medially [...]'.

We thank the reviewer for the suggestion. We compared the absolute values of the MNI *x*-coordinates and found that channels encoding semantic and memorability features had significantly smaller *x*-values than those encoding visual features. However, this difference was mainly driven by the areal distinction

between the VTC and MTL and was therefore trivial. Accordingly, we have removed this argument from the revised manuscript and focused instead on the areal differences in the encoding of different features.

- On page 21 of the discussion, the ATL is mentioned in detail, but it is unclear whether the authors are describing results from the current or a previous manuscript as the ATL is not mentioned in depth in the manuscript itself.

We thank the reviewer for pointing this out and apologize for the confusion. We initially discussed the anterior temporal lobe (ATL) because (1) it is known to be involved in semantic memory, and (2) a key advantage of iEEG is its ability to record ATL responses, which are often inaccessible in fMRI studies due to signal drop-off. However, since our present results are not directly related to the ATL, we have removed these references to keep the manuscript more concise.

Reply to comments from Reviewer 2

This study uses a combination of intracranial recordings and 7T fMRI during a visual task with object images to investigate the representations of visual, semantic, and memorability features across the brain. They find some interesting results, such as more visual features in the VTC, more semantic features in the MTL, and more memorability features in the PFC. They also find PFC memorability representations may emerge from integration in the VTC. However, while the scope of the data is impressive and there are some interesting results, I'm struggling with seeing the bigger picture in the current work. The impact or contribution to current theories is unclear.

We thank the reviewer for the expert and constructive comments.

Major comments:

- The introduction is incredibly short and leaves us with big questions about how the goals of the current work aim to fit in with our current understanding of vision / memory and these brain regions. There have been many studies that have compared DNN representations to brain representations-- for some examples in the memory domain: Davis, Geib, Wing, et al. 2021, Cerebral Cortex; Liu, Zhang, Yu et al., 2021, Science Advances; and then tons in the visual domain, see Cichy & Kaiser 2019 review article. So it seems like one of the major aspects of novelty here is the memorability angle. However, the paper doesn't clearly define what memorability is. Why would this observation of some images being more memorable than others lead to us thinking of it as some sort of attribute represented separately in the brain? How does it relate to more traditional views of visual memory in the brain? Why would the brain be sensitive to an image's memorability? While prior work has shown that AlexNet shows some good correspondences to brain representations, how do we know we can compare ResMem's features to the brain? Further, the paper is missing coverage of several neuroscience studies with memorability that have looked at the VTC, frontal cortex, and MTL: Bainbridge, Dilks, Oliva, 2017, NeuroImage; Bainbridge & Rissman, 2018, Scientific Reports; Mohsenzadeh, Mullin, et al., 2019, Scientific Reports; Jaegle, Mehrpour et al., 2019, eLife; Xie, Bainbridge, Inati et al., 2020, Nature Human Behaviour; Deng, Beck, Federmeier, 2024, Imaging Neuroscience. How does the current work relate to that prior

work? At the same time, it's also not clear how one defines "semantic" versus "visual" features too, and what our current understanding on these two types of features are, too.

We thank the reviewer for the suggestions and appreciate the important outstanding questions and references suggested by the reviewer. In the revised manuscript, we have included an in-depth introduction to better situate our study within the existing literature, clarify the conceptual framework (with a focus on memorability, which is now clearly defined), and highlight the advances of our study:

"A remarkable capacity of the human brain is its ability to encode and retain detailed visual memories after just a single exposure [31-33]. However, the neural mechanisms that support such vivid memory formation remain incompletely understood. The exceptional ability relies on the integration of visual and semantic processing, which interacts with the intrinsic memorability of object images. The concept of "memorability" arises from the observation that certain images are consistently more memorable than others, even within the same category [34, 35]. This effect is robust across individuals and constitutes an isolable phenomenon that cannot be explained solely by attention, semantics, or low-level visual features [26, 36, 37]. Recently, understanding variations in the memorability of visual images has garnered increasing attention, as it is critically linked to visual memory and the development of reliable measures for predicting memory performance [37, 38].

Neural correlates of memorability have been identified in multiple brain areas, including the ventral temporal cortex (VTC), superior temporal cortex (STS), medial temporal lobe (MTL), and parietal cortex [39-43]. Notably, highly memorable images are more decodable and elicit stronger and more persistent neural responses than low-memorability images [41, 43, 44], suggesting that memorability facilitates perceptual processing and thus contributes to better memory performance. Importantly, neural substrates of memorability are dissociable from those of individual memory, indicating that the brain may represent memorability as a distinct attribute—separate from whether a specific image is remembered—within partially independent perceptual and mnemonic streams [39, 41, 45]. Such a representation could confer adaptive value: by preferentially enhancing processing of intrinsically memorable stimuli, the brain may optimize storage of information that is statistically more likely to be behaviorally relevant or socially shared. Our recent study has shown that single neurons in the human MTL not only demonstrate memory selectivity (i.e., differentiating novel versus familiar stimuli) but also correlate parametrically with the memorability scores of images [23], reinforcing the view that

memorability is an intrinsic, image-dependent property with a dedicated neural signature. Together, these findings support the idea that memorability is not merely an epiphenomenon of perception or memory, but a neural feature with functional significance for visual memory systems.

Despite these findings, it remains unclear what makes some images more memorable than others [36] and how memorability interacts with perception and memory at the neural level. Earlier studies primarily linked the memory fate of stimuli to attentional states and task context, utilizing individual-centric methods [46-48]. However, recent advances in deep learning algorithms and the availability of large-scale online behavioral studies have facilitated stimulus-centric research, revealing that visual and semantic features embedded in object images can predict memorability scores [49-51]. Recent research has demonstrated that memorability is represented in both higher visual cortex and mnemonic regions, where it may interact with high-level visual and semantic factors [43, 52]. However, it remains unknown whether memorability is represented as a distinct neural code or emerges from the interplay of visual and semantic processing. In particular, the mechanisms through which the neural processing of the three interrelated attributes—visual, semantic, and memorability—culminates in the formation of visual memory have yet to be elucidated. Specifically, the higher visual cortex subserves the neural representations of visual [53], semantic [54], and memorability [43] features. Are these representations independent of one another, or are they interconnected within the spatiotemporal dynamics of the higher visual cortex and other associated brain regions? Elucidating these questions requires high spatiotemporal resolution signals to enable fine-grained analysis within specific brain areas, particularly the anterior ventral temporal cortex and MTL, which are prone to signal loss in neuroimaging studies [55, 56]."

- And then relatedly, it was hard to read through the findings and grasp the big picture from them--what is our updated model of visual memory / perception, given these results? Part of the problem is that it was hard to know how to interpret seemingly contradictory findings (does memorability have representations in the MTL or not?), and there was a big chunk of results with the 7T data where memorability was not analyzed or mentioned at all ("Validation and generalization with high-resolution 7t fMRI data").

We thank the reviewer for raising this important question. To better clarify the big picture and conceptual advances of our study, we have explicitly articulated our results in the revised manuscript. Specifically, by comprehensively and systematically characterizing the spatiotemporal patterns of neural encoding for visual, semantic, and memorability attributes, we argue that memorability is encoded in a distributed manner in the brain, which can be dissociated from visual and semantic coding yet integrates visual and semantic information.

We apologize for the confusion, but we have now included the analysis of memorability using the 7T fMRI data (**Fig. 4K-O**). Consistent with the iEEG results from the ImageNet (**Fig. 2**) and COCO (**Fig. 5**) datasets, as well as the results from single-neuron recordings (**Fig. 6**), we observed memorability coding in the MTL (**Fig. 4K-O**). We also found that semantic coding was stronger than memorability coding in the MTL, which aligns with our iEEG results. Together, while we acknowledge that different measurement modalities may reveal complementary yet distinct signals, we observed consistent results across all modalities for the encoding of memorability in the MTL.

We have included the following results in the revised manuscript:

"In addition, although we also observed robust encoding of memorability features in both the FG and MTL (**Fig. 4K**), model coding strength was significantly greater for visual features in the FG (**Fig. 4L**; $t(7) = 7.55$, $P = 1.31\times10^{-4}$) and for semantic features in the MTL (**Fig. 4M**; $t(7) = 28.23$, $P = 1.79\times10^{-8}$), consistent with our iEEG results above."

And:

"Similarly, for memorability features (**Fig. 4N, O**), the percentage of memorability-coding iEEG channels that overlapped with memorability-coding fMRI voxels was higher than that of non-memorability-coding channels in the FG ($P = 0.0042$; but not in the MTL: $P = 0.49$). As expected, we further confirmed greater model coding strength in the fMRI-overlapping iEEG channels that significantly encoded visual (**Fig. 4D, E**; two-tailed two-sample *t*-test: FG: $t(108) = 12.44$, $P = P < 10^{-20}$; MTL: $t(99) = 12.49$, $P < 10^{-20}$), semantic (**Fig. 4I, J**; FG: $t(108) = 13.73$, $P < 10^{-20}$; MTL: $t(99) = 10.53$, $P < 10^{-20}$), and memorability (**Fig. 4N, O**; FG: $t(108) = 13.80$, $P < 10^{-20}$; MTL: $t(99) = 10.96$, $P < 10^{-20}$) models, compared to those fMRI-overlapping iEEG channels that did not encode these models."

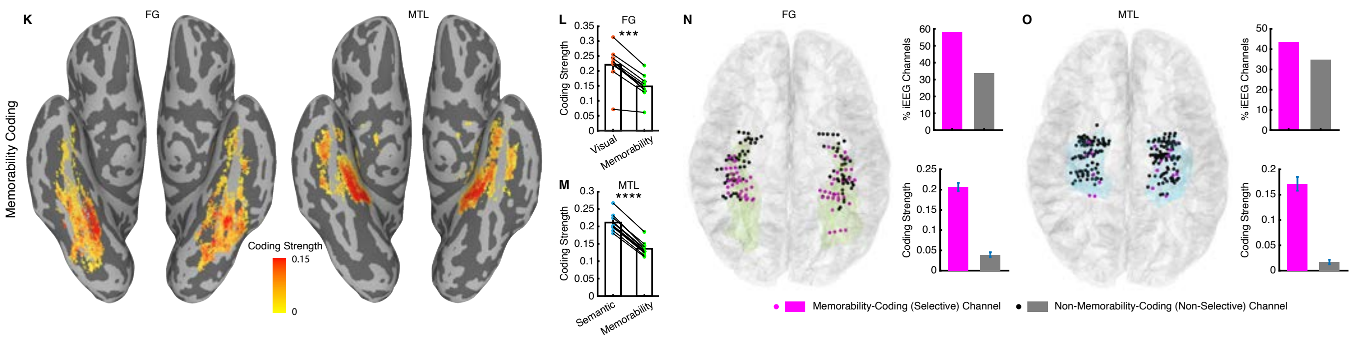

Fig. 4. Validation and generalization with high-resolution 7T fMRI data. (A-C, F-H, K-M) Encoding of visual, semantic, and memorability features using 7T fMRI data. **(A)** Visual coding. **(F)** Semantic coding. **(K)** Memorability coding. Color coding indicates model coding strength, estimated by the Pearson's correlation between the observed and predicted responses. (left) Fusiform gyrus (FG). (right) Medial temporal lobe (MTL). **(B, C, G, H)** Comparison between visual and semantic coding. **(L)** Comparison between visual and memorability coding. **(M)** Comparison between semantic and memorability coding. **(B, G)** Percentage of significant voxels encoding visual or semantic models. **(C, H, L, M)** Average model coding strength for visual-coding, semantic-coding, or memorability-coding voxels. Each dot represents a single participant, and error bars denote ±SEM across participants (n = 8). Asterisks indicate a significant difference using a two-tailed paired t-test across participants. **: $P < 0.01$, ***: $P < 0.001$, and ****: $P < 0.0001$. **(D, E, I, J, N, O)** Alignment of the fMRI and iEEG results. **(D, E)** Visual coding. **(I, J)** Semantic coding. **(N, O)** Memorability coding. **(D, I, N)** FG. **(E, J, O)** MTL. (left) Spatial alignment of iEEG channels with significant fMRI voxels encoding visual, semantic, or memorability features. (top right) Percentage of iEEG channels overlapping with significant fMRI voxels among selective (i.e., visual-coding, semantic-coding, or memorability-coding; magenta) versus non-selective (non-visual-coding, non-semantic-coding, or non-memorability-coding; gray) channels. (bottom right) Model coding strength of iEEG channels overlapping with significant fMRI voxels, comparing selective versus non-selective channels.

Other comments:

- You mention "visual, semantic, and memorability features were preferentially encoded in distinct brain regions", but at this point you have not shown significant differences between the ROIs, so it's not clear to me that you can make the preferential encoding claim.

We thank the reviewer for raising this point and we have revised this claim as follows:

"Together, by examining the neural correlates of these three attributes collectively and comprehensively, we revealed their differential distributions in the human brain: visual information is represented primarily in the VTC, semantic information is represented mainly in the VTC and MTL, and memorability processing engages a broader distribution of higher-level brain areas beyond the VTC."

- Why were memorability features not looked at in the 7T data?

We apologize for the confusion, but we have now included the analysis of memorability using the 7T fMRI data (**Fig. 4K-O**). Please refer to our response to the question above.

- Is there any memory performance behavior from these participants? How do we know that the memorability features here have any relationship to actual memory encoding / retrieval?

We thank the reviewer for pointing this out. While we did not measure memory performance in the present study, we previously confirmed that memorability scores estimated from this DNN are highly correlated with human memory performance [23]. Furthermore, the relationship between memorability scores or features and memory performance has been validated in numerous prior studies [18, 26, 36, 37, 57]. Notably, the ResMem model [12, 26] that we used to extract memorability features was trained and evaluated with large-scale human behavior data measuring memory performance. We have included the following discussion in our revised manuscript:

"In this study, we did not implement a memory task, and hence no direct predictor of individual memory was used to model neural responses. However, in our previous study, which included a memory task, we showed that memorability scores estimated from this DNN are highly correlated with human memory performance [23]. Furthermore, the relationship between memorability scores or features and memory performance has been validated in numerous prior studies [18, 26, 36, 37, 57]. These previous studies support our current findings using predicted memorability scores."

- The discussion mentions the ATL but I don't see it mentioned in the results at all

We thank the reviewer for pointing this out and apologize for the confusion. We initially discussed the anterior temporal lobe (ATL) because (1) it is known to be involved in semantic memory, and (2) a key advantage of iEEG is its ability to record ATL responses, which are often inaccessible in fMRI studies due to signal drop-off. However, since our present results are not directly related to the ATL, we have removed these references to keep the manuscript more concise.

- It seems like here the MTL is defined as the hippocampus and amygdala. However we might anticipate these two regions to have very different relationships to memory / memorability. What happens if you separate them?

We thank the reviewer for pointing this out. In a previous study using the same object stimuli [23], we separately analyzed the amygdala and hippocampus with respect to memory and memorability and found that both regions exhibited similar response patterns. Based on this similarity, and to increase statistical power, we combined the two regions as the MTL in the present study. We acknowledge, however, that these regions may support different aspects of memory processing. We have included the following discussion in the revised manuscript:

"Furthermore, in the present study, we defined the MTL as encompassing both the hippocampus and amygdala. While these structures may contribute differently to memory processes, our previous study using the same object stimuli [23] found that both regions exhibited similar response patterns with respect to memory and memorability. Based on this similarity, and to maximize statistical power, we combined them into a single MTL ROI. Nonetheless, we acknowledge that the hippocampus and amygdala may support distinct aspects of memory processing, and future work with larger datasets will be necessary to dissociate their respective contributions to perceptual memorability."

- The discussion mentions these results provide evidence against an idea of specialized brain areas for visual / semantic / memorability processing, but has anyone actually claimed that (especially I don't think memorability work claims this!)

We thank the reviewer for this important clarification. Our intention was not to suggest that prior work has explicitly claimed the existence of specialized, non-overlapping brain areas solely dedicated to visual, semantic, or memorability processing—particularly in the case of memorability, where the literature generally supports the idea of distributed representation and intrinsic stimulus-driven effects. Rather, our point was to emphasize that our results support a distributed and overlapping coding scheme in the brain, where individual regions, including those traditionally associated with visual or semantic processing, also encode memorability-related information. We have revised the **Discussion** to clarify this point and avoid suggesting a strawman argument. Our findings align with and extend the view that cognitive attributes such as memorability emerge from interactions across multiple representational levels, rather than being localized to a dedicated module. We have revised the following discussion:

"That is, processing different attributes embedded in object images—such as visual, semantic, and memorability—involves a distributed network, including those traditionally associated with visual or semantic processing. Specifically, these processes interact early in the visual cortex, enabling efficient object representations that extend beyond categorical distinctions to include semantic relationships and memory."

- I don't think it makes sense to average the brain responses to the repetitions of the NSD dataset images, since people's memory experiences will have differed across the repetitions (so familiarity will play a larger role in later repetitions). This may dilute any sort of "memorability" signal.

We thank the reviewer for raising this important question. We agree that, in principle, repetition could modulate neural responses, especially through familiarity or adaptation effects. However, analyses of the NSD dataset have shown that the neural response to individual images remains relatively stable across repetitions, evaluated by calculating noise ceiling [22]. In particular, the average noise ceiling in the visual cortex was 36% (equivalent to a prediction accuracy of $r = 0.60$ in terms of Pearson's correlation), indicating high consistency across repetitions. Therefore, averaging across repetitions does not significantly dilute the underlying signal. Moreover, memorability is increasingly understood as an intrinsic property of images that elicits consistent neural responses across individuals and contexts, independent of viewing history [26, 35, 39]. Specifically, it was demonstrated that repeated presentation of visual images was not related to memorability [26]. From this perspective, repetition is unlikely to

alter the neural encoding of memorability itself, but may instead reflect downstream memory-related processes. Therefore, averaging across repetitions helps improve the signal-to-noise ratio while preserving the stimulus-driven memorability signal. We have added clarification to the manuscript to address this point:

"We averaged neural responses across repetitions of the same image to improve signal-to-noise ratio. While repetition can, in principle, influence neural responses due to familiarity or adaptation effects, prior analyses of the NSD dataset have shown that response patterns remain relatively stable across multiple presentations of the same image [22]. Moreover, memorability is considered an intrinsic property of images that evokes consistent neural responses across individuals and viewing experiences [26]. As such, repetition is unlikely to fundamentally alter the encoding of memorability itself, but may instead influence later memory retrieval processes. Thus, averaging across repetitions allows us to capture a more robust measure of the stimulus-driven neural signal without significantly diluting the memorability-related response."

- I don't quite understand the stimulus selection - why randomly select 100 images for each object category, rather than in a regimented way (e.g., picking images that capture the broader features spaces, like those that vary most in memorability)

We thank the reviewer for pointing this out. Our goal in randomly selecting 100 images per object category was to avoid potential selection bias and to allow for a representative sampling of the natural variability within each category. Importantly, because the source dataset is large and diverse, random sampling inherently captures a broad range of visual and semantic features (see [58] for our previous detailed analysis of this set of stimuli), including variation in memorability. Indeed, we confirmed that the randomly selected stimuli span a wide distribution of memorability scores and other feature dimensions. This approach ensures generalizability of our findings across the natural variability present in each category, rather than restricting the analysis to a specific subset of stimuli defined by one feature (e.g., memorability extremes). We have added clarification to the **Methods** section to better explain our stimulus selection strategy:

“It is worth noting that this random sampling strategy was chosen to avoid potential selection bias and to maintain the ecological validity of the stimulus set. Importantly, the natural variability present in the source dataset means that random sampling inherently captures a wide range of image features, ensuring broad and representative coverage of visual, semantic, and memorability variability within each object category (see [58] for detailed analysis). We verified that the selected stimuli spanned a wide range of visual, semantic, and memorability changes, supporting the generalizability of our results. Therefore, this approach allowed us to examine neural encoding in response to stimuli that reflect real-world variation, rather than restricting analyses to handpicked exemplars based on specific criteria.”

- Why would you expect to see a "memorability signal" in the brain in a one-back task that doesn't really tax working or long-term memory?

We thank the reviewer for this insightful question. While the one-back task does not explicitly engage long-term memory retrieval or require intentional memorization, prior work has shown that memorability is an intrinsic property of visual stimuli dissociable from memory and influences neural processing even during tasks unrelated to memory [39, 45]. In particular, memorability has been linked to automatic encoding processes that occur early during perception, without conscious effort or intention to remember [26]. We have included the following discussion in the revised manuscript:

“Furthermore, the relationship between memorability scores or features and memory performance has been validated in numerous prior studies [18, 26, 36, 37, 57].”

- What was the distribution of memorability in the stimuli? What was the spread for the top and bottom 50 images?

We have now included the distribution of memorability scores in the revised manuscript (**Fig. S1D, E**; see also [23]). We have also marked the spread of memorability scores for the top and bottom 50 images.

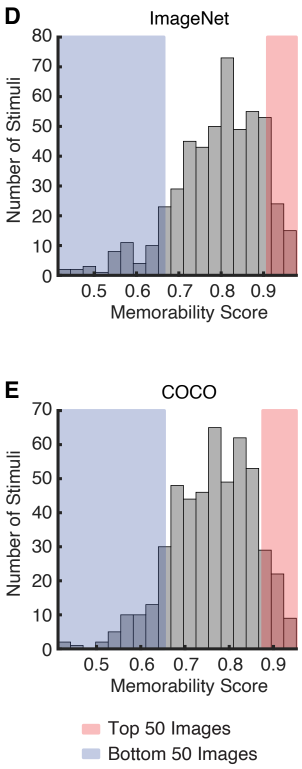

Fig. S1. (D) Distribution of memorability scores for the ImageNet stimuli. **(E)** Distribution of memorability scores for the COCO stimuli.

- *What distance measure was used to make the RDMs?*

We used Euclidean distance for RDMs (please refer to our methods) and we have replicated our results using cosine distance (**Fig. S2G-I**). Please also refer to our response to the third question from Reviewer 1.

- *It would probably be good to clarify that your RSA was done with Spearman correlations only on a triangular half of the RDMs (not the full RDMs I hope)*

We thank the reviewer for the suggestion. We have clarified this in the revised manuscript:

<u>"To compare representations between ROIs, we computed Spearman correlations between their neural RDMs using the upper triangular portion of each matrix."</u>

- For the neural-based RDMs for each channel, what composed the vectors going into the Euclidean distance calculations? (Or was it just a single number like firing rate?)

For each channel, we used the mean HGP (a single number) within a time window of 0.1 to 0.6 s after stimulus onset to compute the Euclidean distances (i.e., absolute differences) for the RDMs. We have clarified this in the revised manuscript:

"Specifically, for individual channels (**Fig. 2B, F, J**), we used the mean HGP (a single value) within a 0.1–0.6 s window after stimulus onset to compute Euclidean distances (i.e., absolute differences) for the RDMs. For population analysis in ROIs with multiple channels (**Fig. 2C, G, K**), we computed the Euclidean distances between neural vectors for the RDMs."

- I don't quite understand the linear regression analysis - it may need more clarification, like showing the actual formulas for the linear models. For example, for the memorability example that only varied along a single dimension of memorability, are you essentially testing if a higher firing rate at one electrode correlates with higher memorability? (And if so, many prior studies have found this sort of effect, so how do these results relate?) Were the 3 types of features ever combined into a single linear model (and if so, there needs to be more methodological detail, like how you balanced different numbers of features)?

We thank the reviewer for these thoughtful questions. To clarify, we have now included the explicit formula for the linear regression analysis in the revised manuscript. Specifically, for each electrode (or voxel), we fit a linear model of the form $\boldsymbol{R} = \boldsymbol{X}\boldsymbol{\beta} + \boldsymbol{\varepsilon}$, where $\boldsymbol{R}$ is the neural response vector, $\boldsymbol{X}$ is the feature matrix (visual, semantic, or memorability features), $\boldsymbol{\beta}$ is the weight vector, and $\boldsymbol{\varepsilon}$ is the error term. This allowed us to assess how well each feature space explained variance in neural responses. We have included the following methods in the revised manuscript:

"We fitted a linear regression model to each channel or voxel to test whether neural representations of visual, semantic, and memorability features could be better explained by a linear model: $\boldsymbol{R} = \boldsymbol{X}\boldsymbol{\beta} + \boldsymbol{\varepsilon}$,

where $\boldsymbol{R}$ is the neural response vector, $\boldsymbol{X}$ is the feature matrix (visual, semantic, or memorability features), $\boldsymbol{\beta}$ is the weight vector, and $\boldsymbol{\varepsilon}$ is the error term."

For memorability, we tested whether neural responses correlated with one-dimensional memorability scores, which effectively reduced to a simple correlation. This analysis replicated previously reported effects showing that more memorable images evoke stronger neural responses, particularly in higher-order regions [39, 40, 45]. We also used high-dimensional features extracted from the ResMem DNN, in parallel with the visual and semantic feature models. Notably, the key conceptual advance of our study is the integration of multimodal data and models to examine how visual, semantic, and memorability features are represented across the cortex with a high spatiotemporal resolution.

Finally, we would like to clarify that we did not combine the three types of features into a single linear model, in order to avoid confounding effects due to feature collinearity and differing dimensionalities. We have clarified all these points in the revised manuscript.

- I don't know how to read Figure 3G. I would overall like to see more visualization of the results from the top-50 and bottom-50 image sets.

We thank the reviewer for the question. **Fig. 3I** (previously **Fig. 3G**) illustrates the neural dynamics of visual and semantic representations for high- versus low-memorability stimuli over time. Specifically, the representational geometry of object images with different memorability scores reveals distinct temporal trajectories following stimulus onset—for both visual features (**Fig. 3I**, left) and semantic features (**Fig. 3I**, right). The separation of the curves indicates that the neural representations of high- and low-memorability images diverge in their temporal evolution, suggesting that memorability modulates the encoding of object features during both early perceptual and semantic processing stages.

In the revised manuscript, we have added two figure panels to visualize the temporal dynamics of the neural responses across visual-selective and semantic-selective channels:

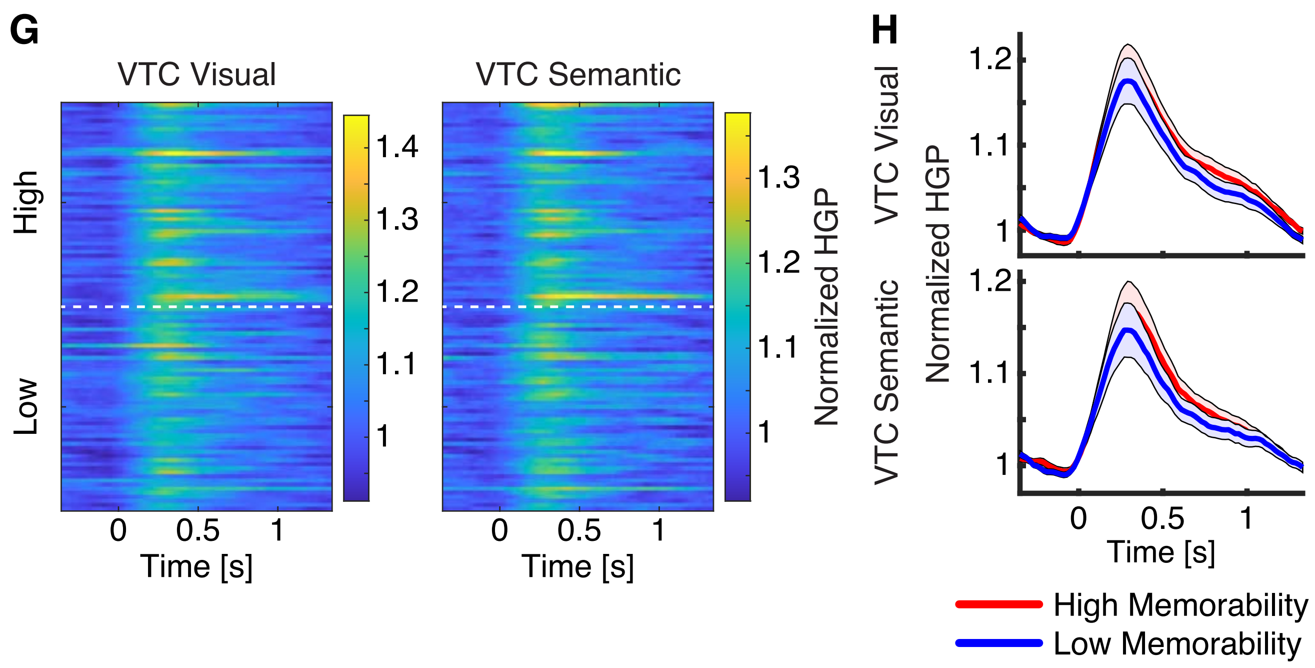

Fig. 3. (G) Normalized high-gamma power (HGP) for object images with high (top 50) versus low (bottom 50) memorability scores. Each row represents an image, sorted by memorability score. Color coding indicates HGP averaged across visual-coding (left) or semantic-coding (right) channels in the VTC. Time 0 denotes image onset. **(H)** Mean normalized HGP for images with high versus low memorability scores. Error bars denote ±SEM across channels.

Notably, we found that, in contrast to the representational geometry (i.e., temporal trajectories), the neural response (i.e., normalized HGP) was similar across stimuli. This indicates that the representational geometry captured additional information that was not evident from the population-averaged neural responses. We have included the following results in the revised manuscript:

"Importantly, in contrast to the representational geometry, the overall neural response was similar across high- versus low-memorability images (**Fig. 3G, H**). This suggests that representational geometry captures additional information beyond that conveyed by population-averaged neural responses."

- I'm a bit confused by Figure 3C - it looks like the time courses don't look so different in terms of peak for the three types of features - is that surprising?

We thank the reviewer for pointing this out. We would like to clarify that we did not assume the three feature types would differ substantially over time. In fact, the time courses for encoding visual and semantic features appeared similar. However, we explored these temporal dynamics and found that semantic encoding peaked slightly later than visual encoding, and memorability encoding was more sustained compared to both visual and semantic features.

- Also some spelling errors I happened to notice:

- *“microwries"*
- *Figure 6: "features form the" (should be "from")*

We thank the reviewer for pointing these out. We have corrected them in the revised manuscript.

Reply to comments from Reviewer 3

This study presents an ambitious and multi-scale investigation of how visual, semantic, and memorability features of natural object images are encoded in the human brain. The authors combine extensive intracranial EEG (iEEG) data, single-neuron recordings, and validation with high-resolution 7T fMRI. They employ representational similarity analysis (RSA) and linear modeling to demonstrate that these three attributes are primarily encoded in distinct but interacting brain regions (VTC, MTL, PFC) and unfold with different temporal dynamics. They further propose that memorability representation arises through integration of visual and semantic information and modulates perceptual processing. This study is an impressive and ambitious effort that combines intracranial EEG, single-neuron recordings, and high-resolution fMRI to address a fundamental question about how visual, semantic, and memorability features are represented in the human brain. Below are my comments for the authors:

We thank the reviewer for the expert and constructive comments.

Please consider discussing implications for how well the DNNs/LLMs capture human visual, semantic and memorability representations.

We thank the reviewer for the suggestion. We have included the following discussion in the revised manuscript:

"Our findings have important implications for evaluating how well current artificial intelligence models—such as DNNs and LLMs—capture human visual, semantic, and memorability representations. While DNNs trained on object recognition tasks often mirror aspects of visual feature coding in the VTC [21, 59, 60], they typically lack representations that correspond to semantic integration and memory-related processes observed in the MTL and PFC. LLMs trained with visual descriptions can capture both semantic relationships based on language structure and visual experiences, as shown in recent publications [61-63]. However, neither the visual DNN models and LLMs inherently model perceptual memorability and they were mostly studied in separate studies. In the present study, our results suggest that human memorability representation arises from the interaction of perceptual and semantic systems and subsequently feeds back to modulate perceptual encoding. This integrative process is not yet well-

captured by current AI models, pointing to the need for future models that incorporate multi-modal learning and memory mechanisms to better reflect human cognitive architecture."

Acknowledge more clearly in the Discussion that this is a correlational neural signature aligned with known memorability predictions, but not a direct predictor of individual memory outcomes in this dataset.

We thank the reviewer for pointing this out. We have included the following discussion in the revised manuscript:

"In this study, we did not implement a memory task and hence no direct predictor of individual memory was used to model neural responses. However, in our previous study, where a memory task was used, we have shown that memorability scores estimated from this DNN are highly correlated with human memory performance [23]. Furthermore, the relationship between memorability scores or features and memory performance has been validated in numerous prior studies [18, 26, 36, 37, 57]. These previous studies back our current findings using predicted memorability scores."

There are many aspects of this study that I appreciate and I consider it to be a strong contribution; however, there are also relevant pieces of literature that should be cited and acknowledged. Some are listed below, but there are many more specially on neural basis of memorability:

Brady, T. F., Konkle, T., Alvarez, G. A., & Oliva, A. (2008). Visual long-term memory has a massive storage capacity for object details. Proceedings of the National Academy of Sciences, 105(38), 14325-14329.

Bainbridge, W. A., Dilks, D. D., & Oliva, A. (2017). Memorability: A stimulus-driven perceptual neural signature distinctive from memory. NeuroImage, 149, 141-152.

Mohsenzadeh, Y., Mullin, C., Oliva, A., & Pantazis, D. (2019). The perceptual neural trace of memorable unseen scenes. Scientific reports, 9(1), 6033.

Jaegle, A., Mehrpour, V., Mohsenzadeh, Y., Meyer, T., Oliva, A., & Rust, N. (2019). Population response magnitude variation in inferotemporal cortex predicts image memorability. Elife, 8, e47596.

We thank the reviewer for the suggestions. We have thoroughly revised the **Introduction** and incorporated these important references. Please refer to our response to Reviewer 2's first question.

References

1. Deng J, Dong W, Socher R, Li L-J, Li K, Fei-Fei L, editors. Imagenet: A large-scale hierarchical image database. 2009 IEEE conference on computer vision and pattern recognition; 2009: Ieee.
2. Ray S, Crone NE, Niebur E, Franaszczuk PJ, Hsiao SS. Neural correlates of high-gamma oscillations (60-200 Hz) in macaque local field potentials and their potential implications in electrocorticography. The Journal of neuroscience : the official journal of the Society for Neuroscience. 2008;28(45):11526-36. doi: 10.1523/JNEUROSCI.2848-08.2008. PubMed PMID: 18987189; PubMed Central PMCID: PMCPMC2715840.
3. Lachaux JP, Axmacher N, Mormann F, Halgren E, Crone NE. High-frequency neural activity and human cognition: past, present and possible future of intracranial EEG research. Progress in neurobiology. 2012;98(3):279-301. Epub 2012/07/04. doi: 10.1016/j.pneurobio.2012.06.008. PubMed PMID: 22750156; PubMed Central PMCID: PMCPMC3980670.
4. Wang S, Tudusciuc O, Mamelak AN, Ross IB, Adolphs R, Rutishauser U. Neurons in the human amygdala selective for perceived emotion. Proceedings of the National Academy of Sciences. 2014;111(30):E3110-E9. doi: 10.1073/pnas.1323342111.
5. Wang S, Mamelak AN, Adolphs R, Rutishauser U. Encoding of Target Detection during Visual Search by Single Neurons in the Human Brain. Current Biology. 2018;28(13):2058-69.e4. doi: https://doi.org/10.1016/j.cub.2018.04.092.
6. Cao R, Lin C, Hodge J, Li X, Todorov A, Brandmeir NJ, et al. A neuronal social trait space for first impressions in the human amygdala and hippocampus. Molecular Psychiatry. 2022;27(8):3501-9. doi: 10.1038/s41380-022-01583-x.
7. Cao R, Wang J, Brunner P, Willie JT, Li X, Rutishauser U, et al. Neural mechanisms of face familiarity and learning in the human amygdala and hippocampus. Cell Reports. 2024;43(1):113520. doi: 10.1016/j.celrep.2023.113520.
8. Cao R, Wang J, Lin C, De Falco E, Peter A, Rey HG, et al. Feature-based encoding of face identity by single neurons in the human amygdala and hippocampus. Nature Human Behaviour. 2025.
9. Cao R, Zhang J, Zheng J, Wang Y, Brunner P, Willie JT, et al. A neural computational framework for face processing in the human temporal lobe. Current Biology. 2025;35(8):1765-78.e6. doi: https://doi.org/10.1016/j.cub.2025.02.063.
10. Mormann F, Kornblith S, Quiroga RQ, Kraskov A, Cerf M, Fried I, et al. Latency and Selectivity of Single Neurons Indicate Hierarchical Processing in the Human Medial Temporal Lobe. The Journal of Neuroscience. 2008;28(36):8865-72. doi: 10.1523/jneurosci.1640-08.2008.
11. Pessoa L, Adolphs R. Emotion processing and the amygdala: from a 'low road' to 'many roads' of evaluating biological significance. Nat Rev Neurosci. 2010;11(11):773-83. doi: http://www.nature.com/nrn/journal/v11/n11/suppinfo/nrn2920_S1.html.
12. Coen D. Needell WAB. Embracing New Techniques in Deep Learning for Estimating Image Memorability. Computional Brain & Behavior. 2022;5:168-84.
13. Aditya Khosla, Akhil S. Raju, Antonio Torralba, Oliva A. Understanding and Predicting Image Memorability at a Large Scale. ICCV2015.
14. Goetschalckx L, Wagemans J. MemCat: a new category-based image set quantified on memorability. PeerJ. 2019;7. doi: ARTN e8169 10.7717/peerj.8169. PubMed PMID: WOS:000502756300004.

15. Muennighoff N. SGPT: GPT Sentence Embeddings for Semantic Search. CoRR. 2022;abs/2202.08904.
16. Simonyan K, Zisserman A, editors. Very deep convolutional networks for large-scale image recognition. International Conference on Learning Representations; 2015.
17. Nils Reimers, Gurevych I. Sentence-BERT: Sentence Embeddings using Siamese BERT-Networks: Association for Computational Linguistics; 2019.
18. Ying Tai, Jian Yang, Xiaoming Liu, Xu C. MemNet: A Persistent Memory Network for Image Restoration. ICCV2017.
19. He K, Zhang X, Ren S, Sun J, editors. Deep residual learning for image recognition. Proceedings of the IEEE conference on computer vision and pattern recognition; 2016.
20. Krizhevsky A, Sutskever I, Hinton GE. ImageNet Classification with Deep Convolutional Neural Networks. NIPS. 2012;1:1097–105. doi: 10.1145/3065386. PubMed PMID: WOS:000402555400026.
21. Bao P, She L, McGill M, Tsao DY. A map of object space in primate inferotemporal cortex. Nature. 2020;583(7814):103-8. Epub 2020/06/05. doi: 10.1038/s41586-020-2350-5. PubMed PMID: 32494012.
22. Allen EJ, St-Yves G, Wu Y, Breedlove JL, Prince JS, Dowdle LT, et al. A massive 7T fMRI dataset to bridge cognitive neuroscience and artificial intelligence. Nature Neuroscience. 2022;25(1):116-26. doi: 10.1038/s41593-021-00962-x.
23. Cao R, Brunner P, Chakravarthula PN, Wahlstrom KL, Inman C, Smith EH, et al. A neuronal code for object representation and memory in the human amygdala and hippocampus. Nature Communications. 2025;16(1):1510. doi: 10.1038/s41467-025-56793-y.
24. Wang J, Cao R, Brandmeir NJ, Li X, Wang S. Face identity coding in the deep neural network and primate brain. Commun Biol. 2022;5(1):611. Epub 2022/06/22. doi: 10.1038/s42003-022-03557-9. PubMed PMID: 35725902; PubMed Central PMCID: PMCPMC9209415.
25. Junnan Li, Ramprasaath R. Selvaraju, Akhilesh Deepak Gotmare, Shafiq Joty, Caiming Xiong, Hoi S. Align before Fuse: Vision and Language Representation Learning with Momentum Distillation. NeurIPS2021.
26. Bainbridge WA. The resiliency of image memorability: A predictor of memory separate from attention and priming. Neuropsychologia. 2020;141:107408. Epub 20200222. doi: 10.1016/j.neuropsychologia.2020.107408. PubMed PMID: 32097660.
27. Cao R, Zhang J, Zheng J, Wang Y, Brunner P, Willie JT, et al. Neural pathway for visual object coding in the human temporal lobe. PsyArXiv. 2024. Epub October 22. doi: https://doi.org/10.31234/osf.io/37epc.
28. Grill-Spector K, Weiner KS. The functional architecture of the ventral temporal cortex and its role in categorization. Nat Rev Neurosci. 2014;15(8):536-48. doi: 10.1038/nrn3747. PubMed PMID: WOS:000339392400009.
29. Grill-Spector K, Weiner KS, Kay K, Gomez J. The Functional Neuroanatomy of Human Face Perception. Annual Review of Vision Science, Vol 3. 2017;3:167-96. doi: 10.1146/annurev-vision-102016-061214. PubMed PMID: WOS:000411715700008.
30. Kanwisher N. Domain specificity in face perception. Nat Neurosci. 2000;3(8):759-63. Epub 2000/07/21. doi: 10.1038/77664. PubMed PMID: 10903567.
31. Standing L. Learning 10,000 Pictures. Quarterly Journal of Experimental Psychology. 1973;25(May):207-22. doi: Doi 10.1080/14640747308400340. PubMed PMID: WOS:A1973P832100006.

32. Biederman I. Recognition-by-components: a theory of human image understanding. Psychological review. 1987;94(2):115-47. doi: 10.1037/0033-295X.94.2.115. PubMed PMID: 3575582.
33. Brady TF, Konkle T, Alvarez GA, Oliva A. Visual long-term memory has a massive storage capacity for object details. Proceedings of the National Academy of Sciences of the United States of America. 2008;105(38):14325-9. Epub 20080911. doi: 10.1073/pnas.0803390105. PubMed PMID: 18787113; PubMed Central PMCID: PMCPMC2533687.
34. Bainbridge WA, Isola P, Oliva A. The intrinsic memorability of face photographs. Journal of experimental psychology General. 2013;142(4):1323-34. doi: 10.1037/a0033872. PubMed PMID: 24246059.
35. Bylinskii Z, Isola P, Bainbridge C, Torralba A, Oliva A. Intrinsic and extrinsic effects on image memorability. Vision Res. 2015;116(Pt B):165-78. Epub 20150320. doi: 10.1016/j.visres.2015.03.005. PubMed PMID: 25796976.
36. Isola P, Xiao J, Torralba A, Oliva A. What makes an image memorable? Cvpr 20112011. p. 145-52.
37. Rust NC, Mehrpour V. Understanding Image Memorability. Trends in cognitive sciences. 2020;24(7):557-68. Epub 20200505. doi: 10.1016/j.tics.2020.04.001. PubMed PMID: 32386889; PubMed Central PMCID: PMCPMC8818973.
38. Khan ZU, Martin-Montanez E, Baxter MG. Visual perception and memory systems: from cortex to medial temporal lobe. Cell Mol Life Sci. 2011;68(10):1737-54. Epub 20110302. doi: 10.1007/s00018-011-0641-6. PubMed PMID: 21365279; PubMed Central PMCID: PMCPMC11115075.
39. Bainbridge WA, Dilks DD, Oliva A. Memorability: A stimulus-driven perceptual neural signature distinctive from memory. NeuroImage. 2017;149:141-52. Epub 20170127. doi: 10.1016/j.neuroimage.2017.01.063. PubMed PMID: 28132932.
40. Jaegle A, Mehrpour V, Mohsenzadeh Y, Meyer T, Oliva A, Rust N. Population response magnitude variation in inferotemporal cortex predicts image memorability. eLife. 2019;8. Epub 2019/08/30. doi: 10.7554/eLife.47596. PubMed PMID: 31464687; PubMed Central PMCID: PMCPMC6715346.
41. Mohsenzadeh Y, Mullin C, Oliva A, Pantazis D. The perceptual neural trace of memorable unseen scenes. Scientific reports. 2019;9(1):6033. Epub 20190415. doi: 10.1038/s41598-019-42429-x. PubMed PMID: 30988333; PubMed Central PMCID: PMCPMC6465597.
42. Xie W, Bainbridge WA, Inati SK, Baker CI, Zaghloul KA. Memorability of words in arbitrary verbal associations modulates memory retrieval in the anterior temporal lobe. Nat Hum Behav. 2020;4(9):937-48. Epub 20200629. doi: 10.1038/s41562-020-0901-2. PubMed PMID: 32601459; PubMed Central PMCID: PMCPMC7501186.
43. Lahner B, Mohsenzadeh Y, Mullin C, Oliva A. Visual perception of highly memorable images is mediated by a distributed network of ventral visual regions that enable a late memorability response. Plos Biol. 2024;22(4). doi: ARTN e3002564 10.1371/journal.pbio.3002564. PubMed PMID: WOS:001194480500001.
44. Deng W, Beck DM, Federmeier KD. Image memorability is linked to facilitated perceptual and semantic processing. Imaging Neuroscience. 2024;2:1-13. doi: 10.1162/imag_a_00281.
45. Bainbridge WA, Rissman J. Dissociating neural markers of stimulus memorability and subjective recognition during episodic retrieval. Scientific reports. 2018;8(1):8679. Epub 20180606. doi: 10.1038/s41598-018-26467-5. PubMed PMID: 29875370; PubMed Central PMCID: PMCPMC5989217.

46. Nosofsky RM. Attention, similarity, and the identification-categorization relationship. J Exp Psychol Gen. 1986;115(1):39-61. doi: 10.1037//0096-3445.115.1.39. PubMed PMID: 2937873.
47. Rugg MD, Mark RE, Walla P, Schloerscheidt AM, Birch CS, Allan K. Dissociation of the neural correlates of implicit and explicit memory. Nature. 1998;392(6676):595-8. doi: 10.1038/33396. PubMed PMID: 9560154.
48. deBettencourt MT, Norman KA, Turk-Browne NB. Forgetting from lapses of sustained attention. Psychonomic bulletin & review. 2018;25(2):605-11. doi: 10.3758/s13423-017-1309-5. PubMed PMID: 28585055; PubMed Central PMCID: PMCPMC5716934.
49. Koch GE, Akpan E, Coutanche MN. Image memorability is predicted by discriminability and similarity in different stages of a convolutional neural network. Learn Mem. 2020;27(12):503-9. Epub 20201116. doi: 10.1101/lm.051649.120. PubMed PMID: 33199475; PubMed Central PMCID: PMCPMC7670863.
50. Hovhannisyan M, Clarke A, Geib BR, Cicchinelli R, Monge Z, Worth T, et al. The visual and semantic features that predict object memory: Concept property norms for 1,000 object images. Mem Cognit. 2021;49(4):712-31. Epub 20210119. doi: 10.3758/s13421-020-01130-5. PubMed PMID: 33469881; PubMed Central PMCID: PMCPMC8081674.
51. Kramer MA, Hebart MN, Baker CI, Bainbridge WA. The features underlying the memorability of objects. Sci Adv. 2023;9(17):eadd2981. Epub 20230426. doi: 10.1126/sciadv.add2981. PubMed PMID: 37126552; PubMed Central PMCID: PMCPMC10132746.
52. Slayton M, Howard CM, Huang S, Hovhannisyan M, Cabeza R, Davis SW. Semantic Dimensions Support the Cortical Representation of Object Memorability. Journal of Cognitive Neuroscience. 2025:1-27. doi: 10.1162/jocn.a.60.
53. Haxby JV, Hoffman EA, Gobbini MI. The distributed human neural system for face perception. Trends in cognitive sciences. 2000;4(6):223-33. PubMed PMID: 10827445.
54. Kumar S, Sumers TR, Yamakoshi T, Goldstein A, Hasson U, Norman KA, et al. Shared functional specialization in transformer-based language models and the human brain. Nature communications. 2024;15(1):5523. Epub 20240629. doi: 10.1038/s41467-024-49173-5. PubMed PMID: 38951520; PubMed Central PMCID: PMCPMC11217339.
55. Axelrod V, Yovel G. The challenge of localizing the anterior temporal face area: a possible solution. NeuroImage. 2013;81:371-80. doi: 10.1016/j.neuroimage.2013.05.015. PubMed PMID: 23684864.
56. Rossion B, Jacques C, Jonas J. The anterior fusiform gyrus: The ghost in the cortical face machine. Neuroscience and biobehavioral reviews. 2024;158:105535. Epub 20240106. doi: 10.1016/j.neubiorev.2024.105535. PubMed PMID: 38191080.
57. Bylinskii Z, Goetschalckx L, Newman A, Oliva A. Memorability: An Image-Computable Measure of Information Utility. In: Ionescu B, Bainbridge WA, Murray N, editors. Human Perception of Visual Information: Psychological and Computational Perspectives. Cham: Springer International Publishing; 2022. p. 207-39.
58. Wang Y, Cao R, Wang S. Encoding of Visual Objects in the Human Medial Temporal Lobe. The Journal of Neuroscience. 2024;44(16):e2135232024. doi: 10.1523/JNEUROSCI.2135-23.2024.
59. Bashivan P, Kar K, DiCarlo JJ. Neural population control via deep image synthesis. Science. 2019;364(6439). Epub 2019/05/03. doi: 10.1126/science.aav9436. PubMed PMID: 31048462.
60. Cao R, Zhang J, Zheng J, Wang Y, Brunner P, Willie JT, et al. A neural computational framework for face processing in the human temporal lobe. Current biology : CB. 2025. Epub 20250320. doi: 10.1016/j.cub.2025.02.063. PubMed PMID: 40118061.

61. Adrien Doerig, Tim C. Kietzmann, Emily Allen, Wu Y, Naselaris T, Kay K, et al. Visual representations in the human brain are aligned with large language models.
62. Colin Conwell, Jacob Prince, George Alvarez, Konkle T. The Unreasonable Effectiveness of Word Models in Predicting High-Level Visual Cortex Responses to Natural Images. 2023.
63. Wang AY, Kay K, Naselaris T, Tarr MJ, Wehbe L. Better models of human high-level visual cortex emerge from natural language supervision with a large and diverse dataset. Nature Machine Intelligence. 2023;5(12):1415-26. doi: 10.1038/s42256-023-00753-y.
