# Supplementary material for: Characterization of the spatiotemporal representations of visual, semantic, and memorability features in the human brain
Source: PLoS Biol. 2026 Jan 20;24(1):e3003614. doi: 10.1371/journal.pbio.3003614 (PMC12851468; doi:10.1371/journal.pbio.3003614)
Supplement: Attachment — Submitted filename: Response to Reviewers_PB 2_v1.pdf [file pbio.3003614.s014.pdf]

Reply to comments from Reviewer 1

In this revised manuscript, the authors have made substantial changes to the presentation, reporting and interpretation of their findings, including new data. These updates have strengthened the authors' claims, however, there remain key issues regarding anatomical and physiological treatment/ interpretation of the data.

Major:

PFC results and nested data: As intracranial data is nested, i.e. contains multiple observations from the same individuals, it is standard practice to deal with this violation of observation independence via mixed effects designs or collapsing observations within individuals. This issue was raised for PFC findings because it was clear that only a subset of data/subjects (10 electrodes) were driving the statistical effect. The authors have responded to these concerns, but I'm not sure the key issue has been adequately addressed. Firstly, I appreciate the authors focused only on responsive electrodes and have added more PFC data. However, of the 59 "added" electrodes (6 subjects), what is important are how many were responsive and made it into the analysis, for which it appears 29 responsive probes were actually added (with 12 total being significant). I might not have followed correctly as the authors didn't make this clear, but only 2 additional/significant PFC electrodes were actually added. Secondly, the authors note that 7/20 subjects contributed significant PFC channels, suggesting the results are not driven by individual differences. However, this is actually the concern being raised, namely that less than half of the sample shows the reported effect, suggesting it's driven by a subset of subjects and doesn't strongly replicate as an observation. In relation to this, I appreciate that the authors have tried alternative statistical methods to suggest that controlling for subject nesting isn't necessary, but this is more a basic principle of statistical testing than an empirical question. A mixed-effects model, with subjects as a random factor, is required to appropriately deal with multiple observations from the same subjects. I do acknowledge that the authors have adjusted the strength of their claims regarding this result. Finally, the authors note some subjects had repeated recording sessions, it's not clear if this means during the same implantation or a second surgery. If this is just a second session during the same invasive monitoring, it should not be treated as new electrodes/observations, simply more trials for those individuals.

Once again, we thank the reviewer for the expert and constructive comments.

We fully agree with the reviewer that the nested nature of intracranial recordings requires statistical approaches—such as mixed-effects modeling or subject-level aggregation—that respect the lack of independence among observations. This approach has been routinely applied in our previous studies (e.g., [1]). Notably, as described in our previous revision, we conducted a generalized linear mixed-effects (GLME) model of the form percentage ~ 1 + (1 | participant) on PFC *visually responsive* channels (as the reviewer noted), consistent with analyses applied to other brain regions. Due to the limited number of samples, this analysis did not yield a significant fixed effect of memorability coding. However, the random effects of participants were not significant, indicating that the results could not be attributed to individual differences.

Importantly, to ensure sufficient statistical power, in the current revision we extended our analysis to include *all* channels using linear mixed-effects models, leveraging the more sensitive, parametric individual-channel RSA correlation coefficient (see **Methods** for RSA) rather than percentage-based measures and explicitly modeling sessions and/or participants as random effects. Using this approach, we observed a significant correlation coefficient for memorability in PFC channels when modeling participants (correlation coefficient ~ 1 + (1 | participant); P = 0.0052), sessions (correlation coefficient ~ 1 + (1 | participant:session); P = 0.0052), and sessions nested within participants (correlation coefficient ~ 1 + (1 | participant) + (1 | participant:session); P = 0.047) as random effects. These results indicate that memorability encoding in the PFC remains significant even after appropriately accounting for multiple observations within participants and sessions. We have included the following results in the revised manuscript:

"To further evaluate whether PFC memorability encoding persists after accounting for the nested structure of the intracranial dataset, we expanded our analysis to include all PFC channels using linear mixed-effects models. This approach uses the continuous individual-channel RSA correlation coefficient (see **Methods**) as a sensitive measure of memorability encoding and explicitly models participants and/or sessions as random effects. Consistent with the above findings, memorability encoding in PFC channels remained significant when modeling participants (correlation coefficient ~ 1 + (1 | participant); P = 0.0052), sessions (correlation coefficient ~ 1 + (1 | participant:session); P = 0.0052), and sessions nested within participants (correlation coefficient ~ 1 + (1 | participant) + (1 | participant:session); P = 0.047) as random effects. Furthermore, both a bootstrapping test and a generalized linear mixed-effects (GLME) model indicated that individual differences in the percentage of memorability-coding PFC

channels were not significant (both Ps > 0.05; see **Methods** for details). Therefore, these results demonstrate that PFC memorability encoding is robust even after appropriately accounting for the nested nature of the data."

Furthermore, we apologize for the lack of clarity in our earlier response:

First, in the previous revision we refined the PFC region of interest (ROI) to focus on subregions within this broad area that exhibited stronger visual responsiveness. After applying this adjusted ROI, the total number of PFC channels increased to 356 with the addition of 59 new channels (previously 214). Of these, 112 channels were visually responsive (previously 83), and among the visually responsive channels, 12 showed significant memorability encoding (previously 8). It is important to note that, due to clinical constraints, PFC coverage was substantially sparser than in the VTC and MTL (**Fig. 1B**).

Second, we fully acknowledge the reviewer's point that the finding that only 7 of 20 participants contributed significant PFC channels indicates that the effect may be driven by a subset of participants rather than reflecting broad replication across the sample. The limited number of responsive PFC channels per participant inherently introduces substantial variability, which can make effects appear sparse or unevenly distributed across individuals. We recognize that this variability, combined with the small number of task-responsive electrodes, complicates population-level inferences and underscores the importance of properly accounting for nested data structures. Accordingly, we applied linear mixed-effects models to all channels to assess memorability encoding in the PFC. Please refer to our response above for details.

Finally, we would like to clarify that the repeated sessions occurred during the same implantation period, typically on separate days, and thus do not represent new electrodes or independent recordings. However, we replicated all analyses using *only the first* recording session from each participant (**Fig. S2M–O**). We believe this approach provides a more appropriate control than collapsing data within individuals, which can result in unequal numbers of trials across participants. As noted in our previous revision and revised manuscript: *"Furthermore, to account for the fact that a subset of patients underwent two recording sessions (**Table S1**), we repeated all analyses using only the first session from each participant and obtained consistent results (**Fig. S2M–O**)."*

Response timing: It is expected that responses in visual regions occur before those in the MTL and PFC, following a wealth of data. The concern raised regarding the timing of responses was about the data itself, rather than methods of response time detection (onset/peak). As is clear from revision Figure 1, mean HGP plots appear highly similar across brain regions, with changes occurring at/before time zero. This suggests that HGP data has been heavily smoothed, and raises concerns for how such data across brain regions can look so similar. While the authors might highlight their focus on latency of the peak response, this is still being estimated from data that suggests brain-wide response onset across regions immediately to stimulus presentation, which makes little physiological sense.

We thank the reviewer again for this important question and apologize for the confusion. The apparent similarity across brain regions in the HGP traces was indeed due to temporal smoothing in the illustrative figures, which is a common practice for visualization purposes. To clarify, we have now included the unsmoothed raw HGP traces for each brain area (**Revision Fig. 1**). As the reviewer anticipated, the onset latency in the ventral temporal cortex (VTC) preceded that in the medial temporal lobe (MTL) and superior temporal gyrus (STG) by approximately 60–70 ms (**Revision Fig. 1**). Notably, because the VTC spans a large region from posterior to anterior portions, we separately analyzed posterior versus anterior VTC (divided at MNI coordinate $y = -40$). This revealed more pronounced effects: the posterior VTC had an onset latency of 91.3 ms, whereas the anterior VTC showed a latency of 127.5 ms (**Revision Fig. 1**). The posterior VTC also preceded the frontal regions, as the reviewer anticipated. Together, these findings are consistent with the reviewer's expectations and complement the analyses presented in the main text. It is worth noting that we will make the peer review history publicly available so that our response letters and supporting data can be transparently accessed alongside the published paper.

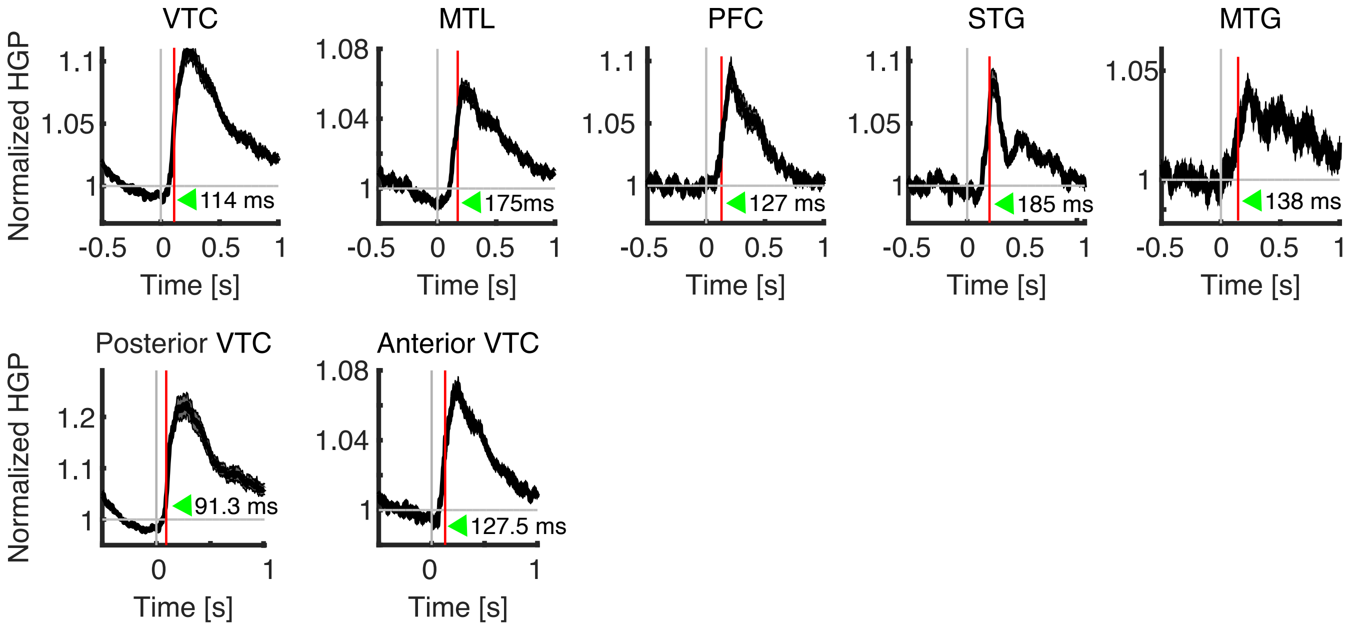

Revision Fig. 1. Unsmoothed high-gamma power (HGP) across brain regions. Shaded areas indicate ±SEM across channels. Green arrows and vertical red bars denote response onset latencies.

Minor:

Model comparison: I appreciate the authors' expanding their modeling analysis. This is very helpful, particularly for the ResNet (visual) and SGPT (semantic) models. However, should we be surprised or read into the finding that multiple layers of SBERT and ResMem outperform or are equal to the final layer in terms of significant channel %? Is that because earlier layers rely more on visual features, so we should expect a greater percentage than later layers?

We thank the reviewer for this insightful question. The observation that multiple layers of SBERT and ResMem outperform or are comparable to the final layer in terms of significant channel percentage is not surprising. As the reviewer correctly noted, earlier and intermediate layers in these models tend to capture lower- to mid-level visual and perceptual features that are more directly aligned with representations in the VTC. In contrast, later layers emphasize more abstract or semantic information, which may correspond less strongly to VTC responses. It is worth noting that consistent with this interpretation, our model comparisons were conducted using VTC responses (**Fig. S2A–E**).

Reply to comments from Reviewer 2

The authors have done a fantastic job at thoroughly addressing my comments and those of the other reviewers. I greatly appreciated the updated introduction that makes the theoretical contribution of the work clearer. The many additional analyses (e.g., using other models, running additional 7T analyses, etc) have also really solidified the strength of the findings. Overall this work will be an important and impactful contribution to the literature and our understanding of how vision and memory work and interact. Great work.

Once again, we thank the reviewer for the expert and constructive comments.

References

1. Cao R, Wang J, Brunner P, Willie JT, Li X, Rutishauser U, et al. Neural mechanisms of face familiarity and learning in the human amygdala and hippocampus. Cell Reports. 2024;43(1):113520. doi: 10.1016/j.celrep.2023.113520.
