# Supplementary material for: Characterization of the spatiotemporal representations of visual, semantic, and memorability features in the human brain
Source: PLoS Biol. 2026 Jan 20;24(1):e3003614. doi: 10.1371/journal.pbio.3003614 (PMC12851468; doi:10.1371/journal.pbio.3003614)
Supplement: Attachment — Submitted filename: Response to Reviewers_PB 3_v1.pdf [file pbio.3003614.s015.pdf]

**Reply to comments from Reviewer 1**

*I appreciate the author's additional responses to the prior round of review, I have no further comments.*

Once again, we thank the reviewer for the expert and constructive comments.
